# Folk classification of wild mushrooms from San Isidro Buensuceso, Tlaxcala, Central Mexico

**DOI:** 10.1186/s13002-020-00408-x

**Published:** 2020-09-14

**Authors:** Roberto Carlos Reyes-López, Adriana Montoya, Alejandro Kong, Ezequiel Alberto Cruz-Campuzano, Javier Caballero-Nieto

**Affiliations:** 1 Posgrado en Ciencias Biológicas, Centro Tlaxcala de Biología de la Conducta, Universidad Autónoma de Tlaxcala, Av. Universidad No 1, Loma Xicohténcatl, 90000 Tlaxcala,, Tlaxcala Mexico; 2 Centro de Investigación en Ciencias Biológicas, Universidad Autónoma de Tlaxcala, Km 10.5 Autopista Texmelucan-Tlaxcala, 90120 Ixtacuixtla, Tlaxcala México; 3 Instituto de Ciencias Biológicas, Universidad de Ciencias y Artes de Chiapas (UNICACH), Libramiento Poniente 1150 Col. Lajas Maciel, 29039 Tuxtla Gutiérrez, Chiapas Mexico; 4 Jardín Botánico, Instituto de Biología, Universidad Nacional Autónoma de México, Tercer Circuito exterior S/N Ciudad Universitaria, Coyoacán, 04510 Ciudad de México, México

**Keywords:** Ethnomycology, Nahuas, Fungi, Macromycetes, Traditional knowledge, Temperate forests

## Abstract

**Background:**

An ethnomycological study was conducted to describe the fungus concept and the traditional fungus classification system for the Nahuas of San Isidro Buensuceso, in central Mexico. The study which provides information on the co-existence of various forms of classification, based on both cultural and biological characteristics.

**Methods:**

The research included conducting community interviews and forest forays in the company of mushroom pickers. The triad technique, pile sorting, and fresh mushroom sampling methods were used. Traditional names were analyzed to describe the Nahua classification system for fungi.

**Results and conclusion:**

The triad technique with non-utilitarian stimuli allowed the fungi to be identified as an independent group of plants and animals. The Nahua people of San Isidro classify fungi primarily based on their use, where they grow, and by humoral characteristics. The analysis of the names revealed a classification based on the criteria proposed by Brent Berlin. This study identified the detailed knowledge of fungi in this Nahua community. The criteria used for the recognition of the species are very reliable, since they use organoleptic, ecological, phenological, and morphological characteristics.

## Introduction

Ethnomycology is an area of Ethnobiology that studies traditional knowledge, manifestations, and cultural and/or environmental implications that derive from the relationship between fungi and man through time and space [1] as well as the mechanisms by which they are generated, transmitted, and evolved in a non-formal way through these dimensions [2]. It emerged as a study area from the 1950s with research on entheogenic fungi and their ritual use in various communities in Mexico, mainly in Oaxaca [3]. Since that time and to date, the development and evolution of the discipline have been dramatically strengthened [4], and the development of specific methods for the exploration of various topics has been of great relevance. From the application of molecular tools for the study and identification of fungi, the little explored high diversity of fungal species used throughout Mexico and the world has become increasingly evident. Native groups living near forests use only a small proportion of this group of organisms [5]. One of the aspects still little explored worldwide is the way in which they are classified by the people of these groups. Based on the results of studies focused on knowing the classification of fungi, it has been seen that they are generally classified based on their use. What is used is mostly named, but also the analysis of the nomenclature allows us to observe a grouping with a structure hierarchy based on what was indicated by Berlin [6]. The importance of analyzing the criteria used to make the groupings is evident. It was necessary to generate information that was more detailed than previous studies to discover the existence either of a general pattern for classifying fungi, or to describe the different ways in which fungi are grouped by each ethnic group or traditional society. The objective of this work is to describe the way mushrooms are conceived, the way they are classified, and the criteria used for this, by the study of ethnomycology in an original Nahuatl community in Central Mexico.

The Nahuas make up the largest ethnolinguistic group in Mexico; they comprise of several groups of people that have language as a common element, but they inhabit different regions of the country, including the state of Tlaxcala, which is the smallest and is located in Central Mexico. There is extensive information about traditional fungal knowledge; however, the Nahua indigenous classification system for fungi has been poorly explored despite the important role it plays in the understanding of the way in which fungi are known and used thereby providing a foundation from which their conservation and sustainability could be managed. Information obtained in different parts of the world about different systems used today do not explain how insufficient to determine how these organisms are grouped or classified by these local people.

There are very few studies of folk classification associated with fungi in Mexico. The first recorded research was with Purepechas from Michoacan. It was observed that mushrooms are included as a life form and are grouped using utilitarian criteria [7]. A folk taxonomic hierarchy with different levels was proposed by the authors. Also in Michoacan state, Aniceto-Crisostomo [8] found that inhabitants of a community from Zitacuraro classified mushrooms using utilitarian criteria. They used three groups: edibles, inedibles which are known to be poisonous (and have traditional names), and others (without names and edibility unknown locally). Mestizos from Ajusco, in the area surrounding Mexico City, use several criteria to classify their mushrooms: ecology, morphology, and edibility. Using such criteria, people group these organisms by the vegetation types and habitats in which they grow [9]. Inhabitants of Pixoy, Valladolid, Yucatan, in Southeast Mexico, classify mushrooms according to where they grow but do not use morphology [10]. The Ocuilteca people from the State of Mexico classify their fungi using three criteria: morphology, phenology, and utilitarian [11]. The Lacandones (a Mayan group) from Lacanjá-Chanzayab in Chiapas, name the mushrooms “*kushum*,” which includes all the known classes of fungi, encompassing all the organisms that cause various materials to rot and this is the characteristic that separates them from plants and animals. It is the most inclusive category and is divided into 21 folk genres [12]. In the case of the Zapotecs of Mixtepec, Oaxaca, Hunn et al. [13] suggest that mushrooms comprise a fungal life form affiliated with neither the plant nor the animal kingdoms. Tzeltal Maya classification (in Chiapas) was described by Lampman [13] and shows a structured system according to the principles proposed by Berlin [6], but he also pointed out the presence of utilitarian criteria.

In other parts of the world, for example between the Chewa from Malawi (Africa), Morris [14–16] showed that the Chewa classification of fungi includes bowa (edible) and chirombo (inedible species) and noted that these two groups are considered different entities. This grouping appears to be influenced more by cultural values than by morphology and this view is supported by the small number of chirombo names (only two species). More examples around the world show that fungi are considered, either as a life form of plants or rarely as part of the animal kingdom, and also as a separate group of animals and plants. Recently, Koyowski et al. [17] carried out a regional ethnomycological study in Mazobia, Poland. They include some data about the folk classification of fungi, which are divided into several groups: the edibles known as gasky (literal geese) due to the similarity of the fruiting body between them; the poisonous or non-edible mushrooms that are known as psiaki or dog mushrooms include all species with small fruiting bodies in the order Agaricales, and the shelf mushrooms that correspond to Polyporales named hubi. The factors used in the folk classification of fungi vary according to the taxa. For order, family, and genus level, the recognition criterion used is the shape of the fruiting body; for the species recognition (in the broad sense)/sections, characteristics such as shape, color, and utilitarian properties of the fungi are taken into account. For the species level (in the strict sense), shape, color, utilitarian properties, symbiotic relationships, habitat, phenology, taste, odor, meat characteristics, and the presence and characteristics of latex are the characteristics used. The authors obtained 526 folk names for fungi and recognized species with many names (e.g., *Leccinum aurantiacum* with 25 different names) and others homogeneously named with one single name throughout the region, as in the case of *Lactarius delicious* also known as rydz. Although this research is important due to its regional nature, the aim was not to obtain the folk classification of fungi. The method was not focused on the subject, yet it allows us to observe the importance of conducting folk classification studies as in this case to show a mixture of utilitarian and morphological criteria for grouping the different fungal taxa.

**Fig. 1:**
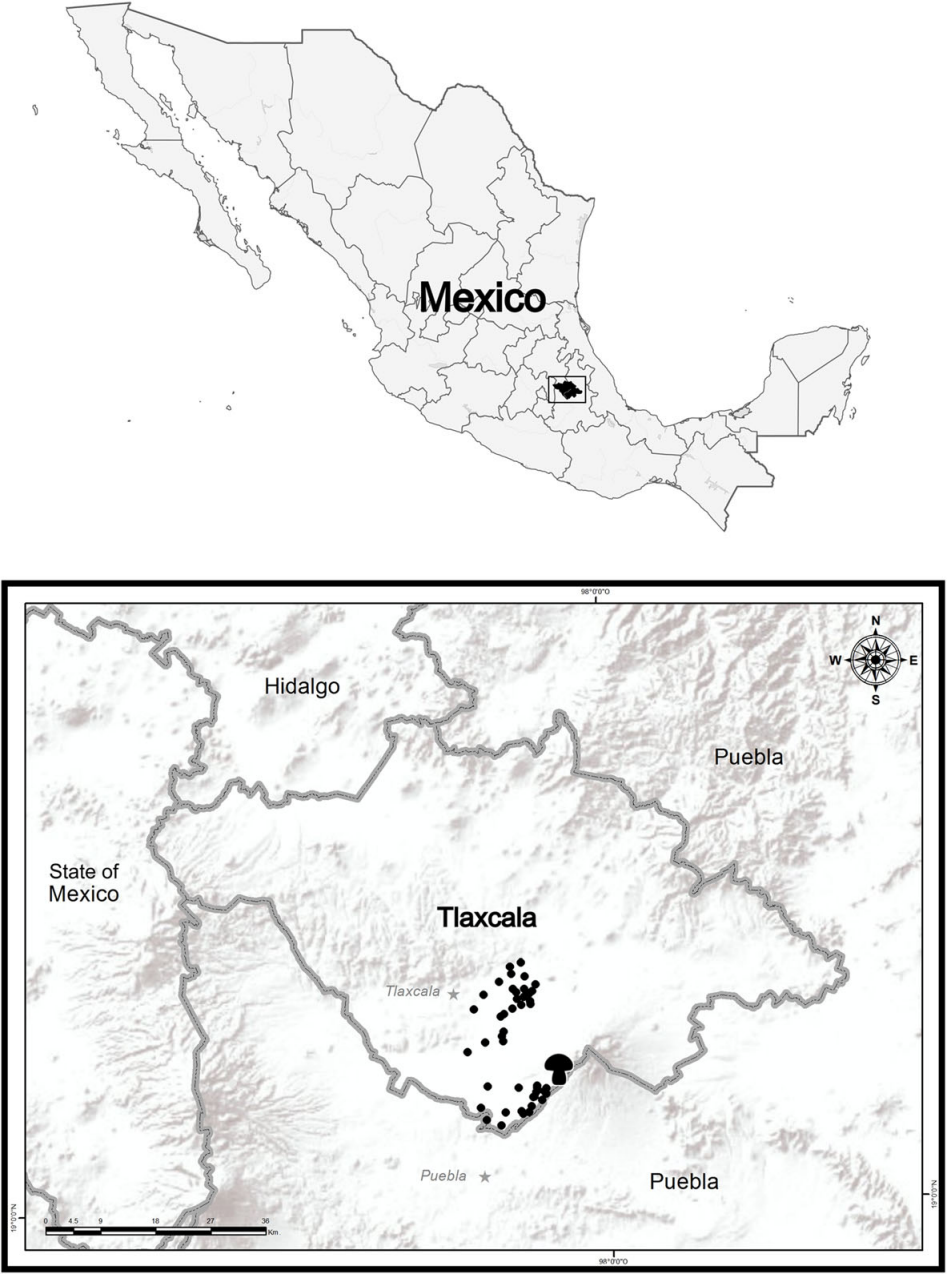
Location of San Isidro Buensuceso, Tlaxcala in Central Mexico. Dots represent the area with Nahua speakers and the small mushroom is in the studied locality

This study explores the use of traditional names and a classification system to aid understanding of the main criteria used by local communities to identify and distinguish between edible and poisonous species of mushrooms. Poisoning from ingesting fungi is a major health issue in central and south Mexico. The findings will increase understanding of species traditional identification and consumption and also generate biocultural information for the conservation and management of the forest of the Malinche National Park, Tlaxcala, where the research is conducted. The research is focused on the mushroom concept and Nahuatl ethnomycological classification used by people from San Isidro Buensuceso (SIBS). The town of San Isidro Buensuceso (2600 m asl) belongs to San Pablo del Monte municipality, in the state of Tlaxcala. It is located on the slopes of La Malintzi volcano (4460 m asl) [18]; this mountain belongs to the Transmexican Volcanic Belt in Mexico (Fig. 1).

According to the data of the Humantla Meteorological Station and using the Köppen Climatic Classification, the type of weather is C (w_2_) (w), sub-humid with the rainy season in summer [19]. The annual mean precipitation varies between 700 and 1000 mm. The rains generally last from June to September but sporadic precipitation can occur for four more months [20]. The annual temperature fluctuates between 12 and 18 °C [19].

The vegetation types reported in the area are fir forests (*Abies religiosa*), pine forests (*Pinus hartwegii*, *P*. *leiophylla*, *P*. *montezumae*, *P*. *patula*, *P*. *pseudostrobus*, *P*. *teocote*) and oak forests (*Quercus rugosa*, *Q*. *crassipes*) [21]. The village is surrounded by temporal farms growing the following principal crops listed in order of importance: corn (*Zea mays*), bean (*Phaseolus vulgaris*), fava bean (*Vica faba*), wheat (*Triticum aestivum*), pumpkin (*Cucurbita* sp.), and fig-leaf gourd (*Cucurbita fisiofolia*) [19].

**Fig. 2:**
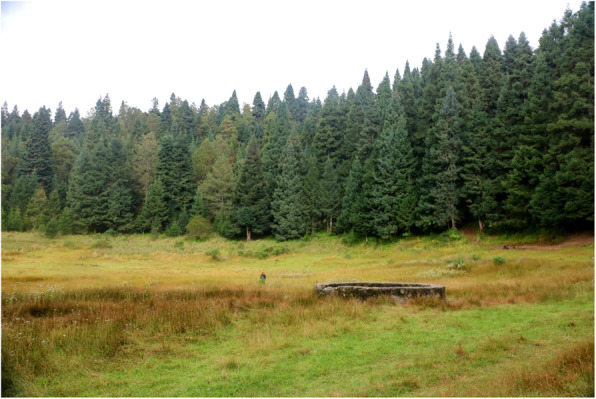
Fir forest in La Malinche National Park where people from San Isidro Buensuceso collect some fungi species. Site of great biocultural significance known as Tlalocan in which rituals and festivals are held to ask for rain

Habitants of SIBS belong to the original Nahua group, which is the most numerous at national scale. Population census effected by INEGI [22] gave the population size of SIBS as 8769 inhabitants of which 4367 are male and 4402 are female. Of those over the age of 5 years, 5896 were indigenous language speakers, 207 being monolingual, and 6407 bilingual [22]. This means that the community of SIBS is among the highest speakers of Nahuatl in the state of Tlaxcala. This Nahua community preserves and utilizes a wide diversity of corn breeds and employs and recognizes through different traditional names the natural resources available from the agroecosystems that the mountain offers [23]. The consumption of wild mushrooms takes place at different periods of the year. Local people collect from agricultural fields, oak forests near the village, and from the different habitats present at La Malinche National Park. They also forage on private property and communal lands (Fig. 2).

## Materials and method

### Field study

This study is part of a long-term ethnomycology research project that started in 2007 and continues to this day in the SIBS community. The information was obtained throughout two rainy seasons and in 2017 the last collections were made. Permission to carry out the study was given by the local authority (the auxiliary presidency of SIBS). During the development of this research project, the participants followed a code of conduct based on the suggestions from the Code of Ethics [24] and Ethics Code of SOLAE [25]. Routes were walked throughout the rainy season covering locations commonly frequented by mushroom collectors accompanied by local experts (named as “hongueros”), mycologists, and students. During the execution of such routes, information about useful and non-useful species was obtained and their common names in Spanish and Nahuatl were recorded. Information regarding the specific places and seasons in which mushrooms grow was also recorded. Mushrooms collected along each route were shown to inhabitants of the community to corroborate the folk nomenclature and also to determine the number and groups of mushrooms that people recognized, as well as the criteria used for groupings and discriminating between mushrooms. Pictures of the collections were taken and used as stimuli in the next part of the study. After the taxonomical determination of the fungi collections, they were deposited in the TLXM Herbarium at the Universidad Autonoma de Tlaxcala. Interviews were conducted at different phases of the investigation, with the cooperation of 30 individuals.

### Fungi concept using the triad method

The triad method consists of presenting items or objects in sets of three to each person. Triadic comparisons can be used to collect similarity or ordered data. In the first case, persons are asked to pick, from each set of three items, the item that is most different from the other two [26]. This technique was applied to understand the mushroom concept and this included the use of photographic stimuli.

**Fig. 3:**
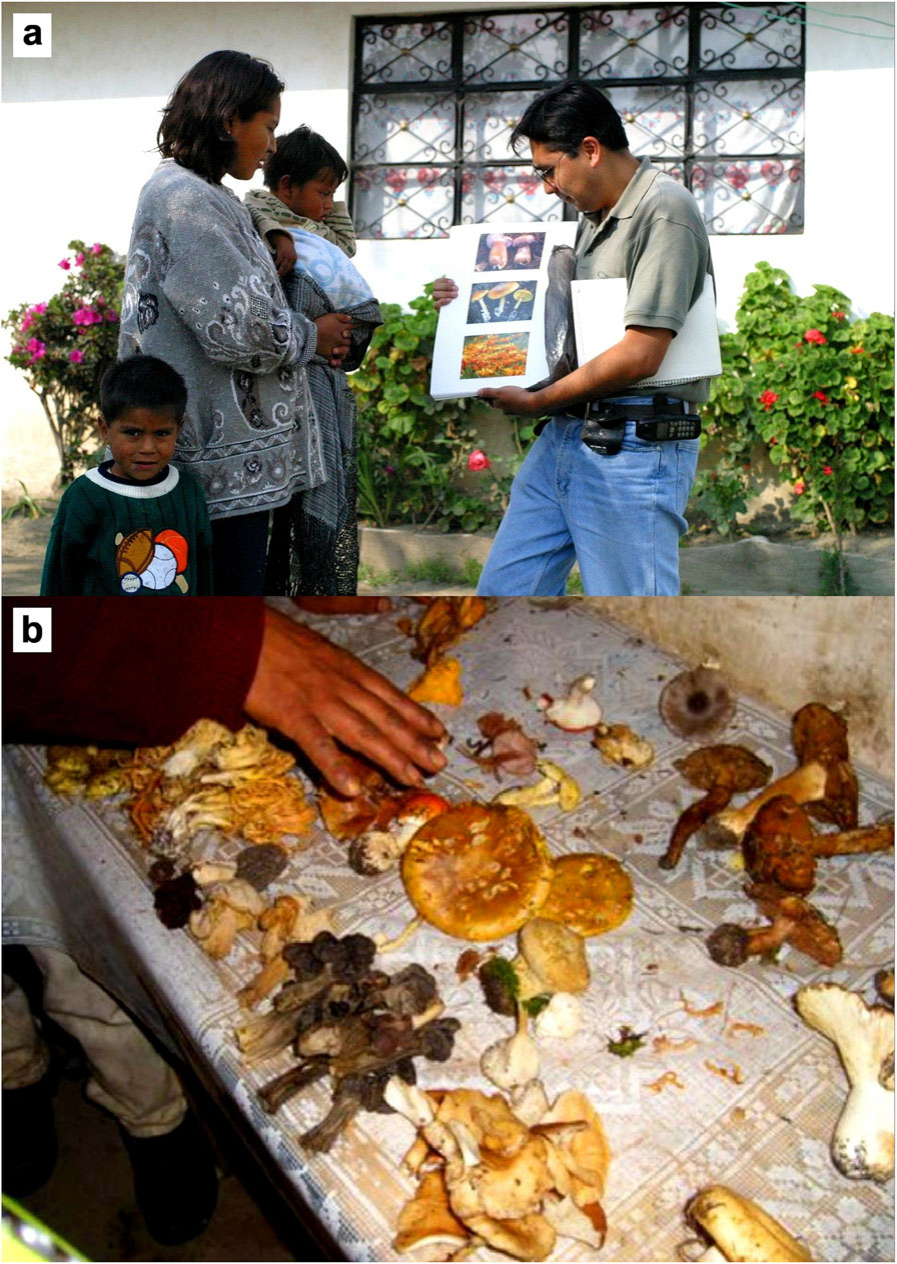
**a** Interviews with Nahua persons from SIBS, Mexico using the triadic technic. **b** Exercise to grouping fresh fungi after collect them in la Malinche National Park

Considering previous ethnomycological studies in which fungi are considered plants, food, fungi, and/or animals [27–31], nine photographs (10 × 10 cm) including fungi (*Phaeolus shwenitzii*, *Amanita muscaria*, *Trametes versicolor*, *Clavariadelphus truncatu*s), plants (*Eucaliptus* sp., *Larrea tridentata*, *Festuca tolucensis*), and animals (*Passerculus sandwichensis*, *Sceloporus aeneus*, *Peromyscus levipes*) from the region were shown in groups of three, mounted on carboards of 45 × 30 cm (Fig. 3a). They were selected taking into account the results of several pilot tests with different organisms and meals in the community; we chose to include non-commonly useful organisms. Photographs bearing organisms showcased the environment in which they were found. Each participant, chosen by random, was asked to pinpoint the two objects that “go together” or “looks more alike” on each sheet and the reasoning for their selection was recorded.

### Traditional classification using the pile sorting technique

In order to determine the traditional classification system, the pile sorting technique [26] was used. It involves successive divisions of formed groups. A total of 82 photographs were utilized (15 × 10 cm). These pictures were exclusively of the mushrooms found in the region, including edibles, medicinal, wood decomposers, and poisonous mushrooms. The images were taken during the ethnomycological forays in the forest with people from the village. The stimuli were shown to 20 individuals (10 specialists in mushroom foraging and 10 people picked at random in the village). Participants were requested to group the photographs according to what they think should go together, meaning that they would pile mushrooms that shared resemblances one over the over to form mounds or piles. Participants were asked to explain the criteria behind each grouping.

To compliment the photographic study, fresh mushrooms were also shown to a group of 14 individuals on four different occasions. They were asked to group the mushrooms according to those that go together and also to explain the reasoning behind such groupings (Fig. 3b). Analysis of the results of these two different grouping studies was used to identify the classification system.

### Linguistics analysis

In order to understand the folk taxonomic categories presented in the Nahua classification of the mushrooms of ethnobiological importance to SIBS, a linguistic analysis of the Nahuatl traditional names given to the mushrooms was made; grammatical categorization of traditional names included all, Spanish and Nahuatl names. In both cases, the principles proposed by Berlin [6] were used.

## Results

### Fungal concept

During discussions with participants in the community, it was found that the use of the term “*nanacatl*” (which means meat, according to Martín del Campo [32]) could initiate a conversation on the topic of interest. This term promptly led to a flux of vernacular names regarding the different constituents of what science has defined as the kingdom of fungi [33].

Some distinctive characteristics of all nanacatl are recognized. The principal of these lies in their temporality, since they can only be found in the rainy season. Some people pointed out that fungi are a product of the earth, while others said that they are produced by God and some denoted that they are produced by seeds and roots or they are born from ocō-xāl (leaf litter of pines and other conifers). It was mentioned that they differ in their color, that the place where they grow varies, and that unlike animals, they do not move. People distinguish them from plants because these are always found; mushrooms appear only in the rainy season when it is possible to find them. They use another term for plants (xihuitl = herbs) and animals are named as the (yolkatl *=* animals).

**Fig. 4:**
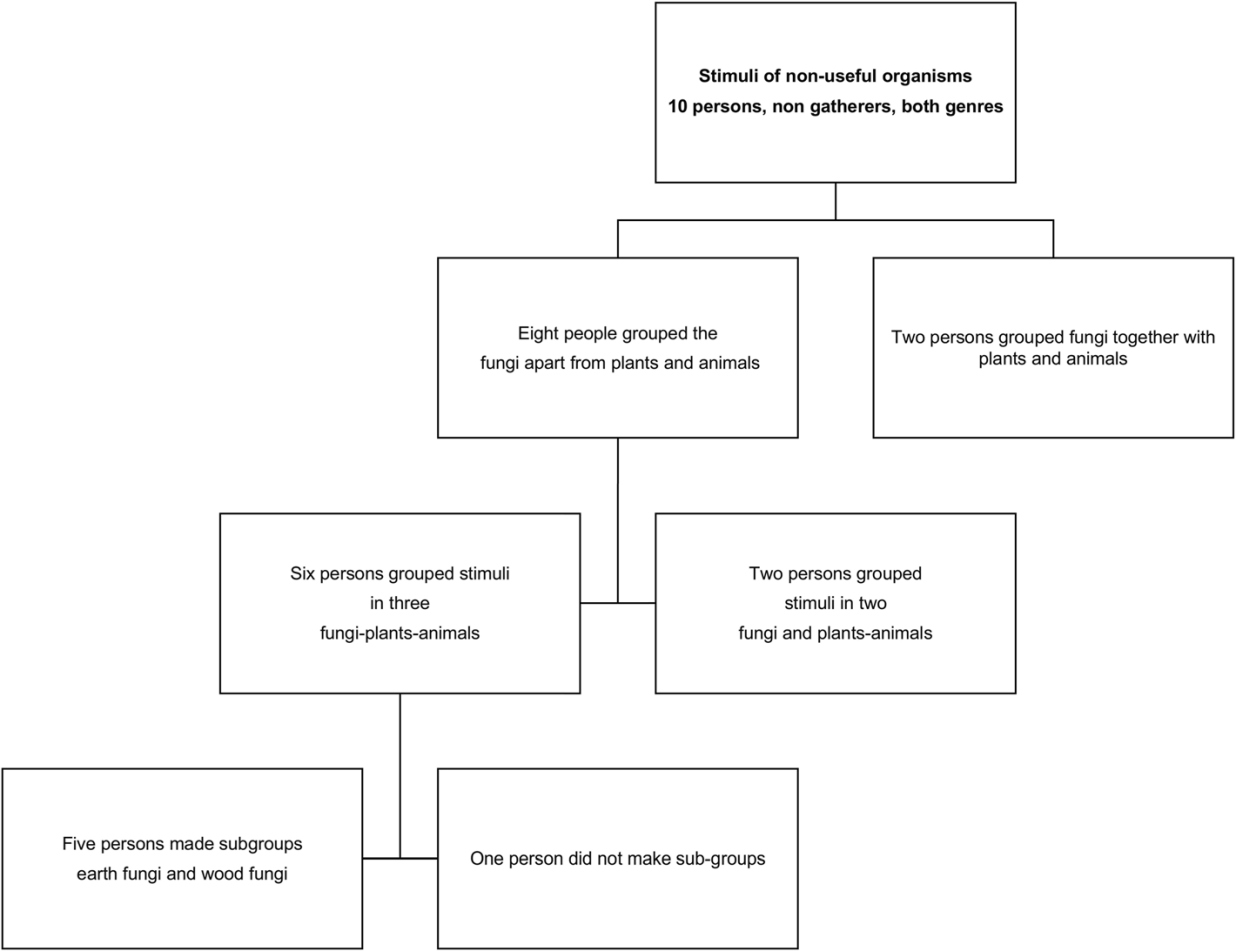
Diagram showing the answers to the fungi concept in SIBS, Mexico

Using triad technique, the concept of fungi as separate entities from plants, animals, and meals varied at first depending on the stimuli shown. The view that they are a meal was present. The latter concept does not detract from the view that they are fungi. Nevertheless, the result was that they are considered as a meal and are named nanacatl (Nahuatl term translated as meat). The criteria for preference in consumption between species are clear as they remark that some mushrooms are really savory, that is they taste like meat. For example, the flavor of āyoh-xōchitl-nanacatl (*Amanita basii*) is like chiken and xō-tomāh (*Boletus edulis* group) tastes like pork. These findings reflect the profound Nahua vision of the environment: fungi are mushrooms; they are edible and taste like meat because of their flavor. Final findings using non-used stimuli showed that fungi are a separate group, that is they are “just fungi” (nanacatl) (Fig. 4). Mushrooms for some people are seen as a natural product that belongs to the “monte” (mountain or forest) and their morphological, ecological, and phenological characteristics are known; some also mentioned their reproduction mechanism mediated by spores (they are born from a white powder), their associations with plants, and their utility for animals. They recognized macroscopic fungi mainly (including polypores-bracket or shelf fungi-). Lichens are not considered fungi, neither are some jelly fungi. Because the best known fungi are macroscopic, people assign names to the structures that make up the basidiocarp of the agaricoid type and some ascocarps (in the case of *Morchella* and *Helvella*). People name the cap (izontecotl = hat), context (ichito = meat), scales (citlal = stars), ring (iyehuayo = skin), sac (iyehuayo = skin), and some of them the spores (seeds, dust) [34].

### Nahua classification of mushrooms

Using the pile sorting technique and interviews with fresh field samples, it was observed that in SIBS, many different ways of classifying fungi co-exist: pragmatic or utilitarian in which nanacatl (fungi) are divided into cuali-nanacatl (good mushrooms) and pitzō-nanacatl (rabies fungi or poisonous).

The majority of cuali-nanacatl have a unique name, whereas the majority of pitzō-nanacatl have names which refer to or are comparable with an edible. People say: “is the double of + the name of the edible mushroom it resembles.” Nonetheless, there are species in this division that hold some importance from a cultural perspective either because their morphological similarity and fructification is in the same location and time frame as an edible or if they have flamboyant morphology and/or outstanding physical-chemical properties (color changing when touched). In most of these cases, the pitzō-nanacatl receive a name that alludes to the name of a look-alike species. They receive the name i-tlatla in (twice as), one example is i-tlatla in tlapīltzal (*Sarcodon* spp. -non edible-), that is the “twice as”, tlapīltzal (*Turbinellus floccosus* -edible-). This is equal to the case of polytypic Purépecha folk generic in Michoacán, México [5]. There are some exceptions to this statement, illustrated with *Amanita muscaria* which is named for its appearance, cītlal-nanacatl (fungi with stars, the cap) and is considered to be the counterpart of āyoh-xōchitl (flower mushroom) (*Amanita basii* -edible-), because of the resemblance between both which makes people link them together and think of them as sisters.

No distinction is made between mushrooms that could cause death and ones that cause different types of intoxication. For example, it is considered by some people that cītlal-nanacatl (*Amanita muscaria*) can cause death if eaten by mistake. It is known from scientific research that consumption of this species causes excitation and depression and affects the central nervous system in a general way [35] and at the same time it provokes gastrointestinal upset [36]. Ramírez-Terrazo [37] proposed a classification for pitzō-nanacatl from SIBS (including three different kinds: (a) poisonous fungi and named, (b) fungi recognized as poisonous and not named, and (c) ignored poisonous fungi). She registered 103 names for non-edibles, and the criteria used to identify each one. Most of the names make reference to the comparable edible species which looks alike and the only one with a special name is *A*. *muscaria*.

**Fig. 5:**
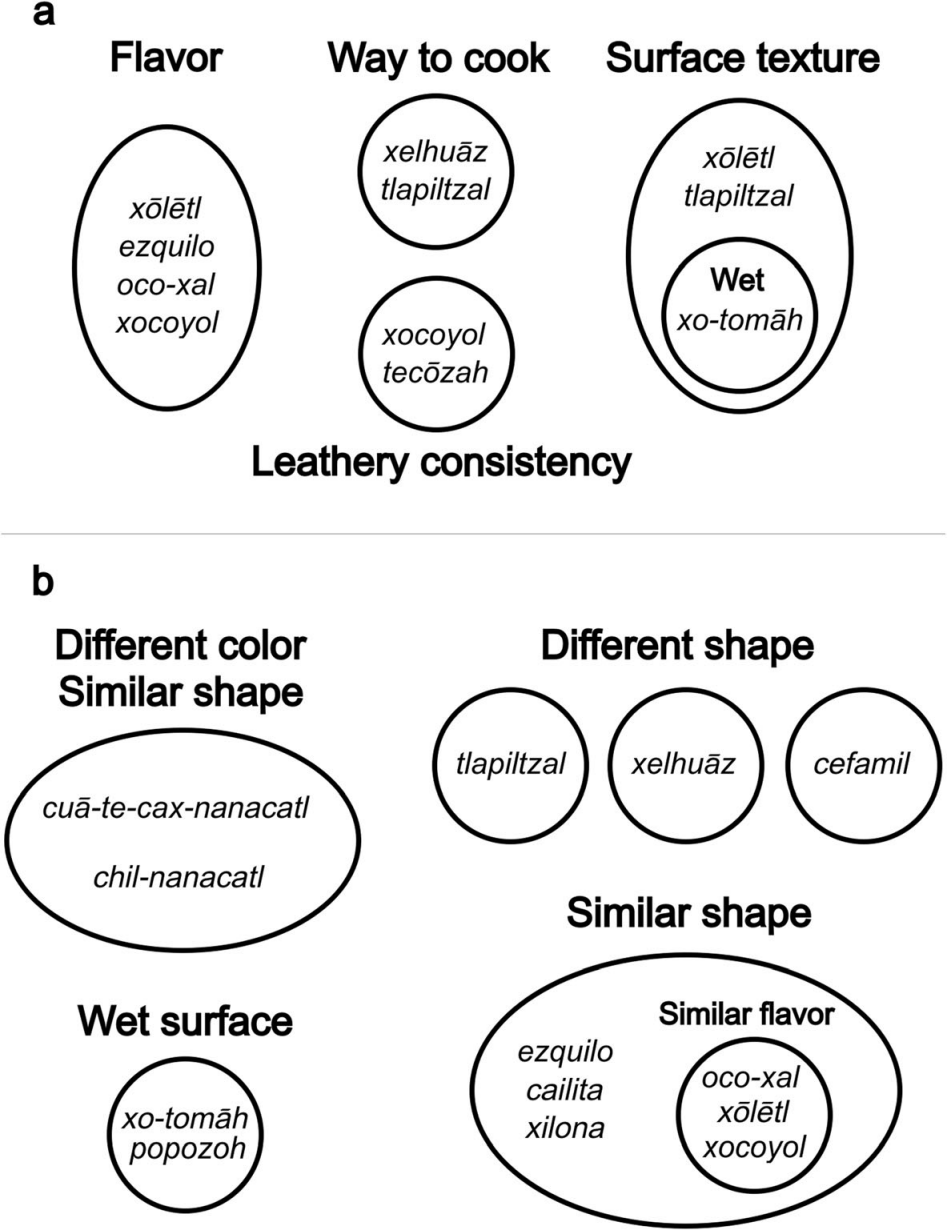
Mushrooms grouping made by Nahua people by different criteria, using pile sorting technique. **a** Shows the grouping of one essay. **b** Shows the second essay

The groupings made by participants in the present study mainly related to their culinary properties (gastronomic criteria) being picked for their consistency and flavor. Mushrooms that were grouped by two people interviewed using fresh samples were as follows: esquilon (*Clitocybe gibba*), ocō-xāl (*Hebeloma* aff. *mesophaeum*), tlalpīltzal (*Turbinellus floccosus*), tecōzah (*Cantharellus* aff. *cibarius*), xelhuāz (*Ramaria* spp.), xocoyuli (*Laccaria bicolor*), and xōlētl (*Lyophyllum decastes*). The grouping is shown in Fig. 5.

On the second occasion, two people were interviewed and the following mushrooms were used: cuah-te-cax (*Russula delica* group), chīl-nanacatl (*Lactarius salmonicolor*), cailita (*Tricholoma equestre*), ocō-xāl (*Hebeloma* aff. *mesophaeum*), ezquilo (*Infundibulicybe gibba*), xocoyuli (*Laccaria trichodermofora*), xō-tomah, (*Boletus pinophilus*), popozoh (*Suillus* sp.), xōlet (*Lyophyllum decastes*), xilona (*Hygrophorus chrysodon*), tlalpīltzal (*Turbinelus floccosus*), xelhuāz (*Ramaria* spp.), and cefamil (*Lycoperdon perlatum*). In this case, persons grouped stimuli as in Fig. 5b.

It was observed that people used different criteria to group mushrooms, such as shape, color, flavor, consistency, and even the form of preparation, combining both organoleptic and culinary criteria.

The third trial was conducted with four people who were shown the following wild mushrooms: xō-tomah (*Boletus pinophilus*), xelhuāz (*Ramaria* spp.), ezquilo (*Infundibulicybe gibba*), xiteburo (*Lycoperdon perlatum*), tecōzah (*Cantharellus* aff. *cibarius*), chīl-nanacatl (*Lactarius salmonicolor*), ocō-xāl-nanácatl (*Hebeloma* aff. *mesophaeum*), xilona (*Hygrophorus chrysodon*), *gā*-*gachupi* (*Helvella crispa*), and ōlō-nanacatl (*Morchella* spp.).

All the fungi were recognized and two people separated them according to the way of cooking them: -In Mole (xelhuāz), in broth with red tomato and garlic (tecōzah, ezquilo), in broth with chilli (the rest).

In another trial, 36 species of fungi were involved and eight people were interviewed. The species used are those in Table 1.

**Table 1:** Categorization of traditional names (in Nahuatl and Spanish) for fungi used in SIBS, Mexico according to Berlin et al. (1992) [6]

Generic taxa designed by simple primary names	
1. amarillitos -little yellow- (*Cantharellus cibarius*)	
2. blanquitos -little white- (*Hygrophorus chrysodon)*	
3. cailita ? (*Tricholoma flavovirens*)	
4. campanilla -beltl- (*Infundibulicybe gibba*, *I*. cf. *squamulosa*)	
5. cefamil ? (*Lycoperdon perlatum*)	
6. cemita .-a kind of bread- (*Boletus atkinsonii*, *Boletus pinophilus*)	
7. clavitos -nails- (*Lyophyllum decastes* complex)	
8. cochinito -little pig- (*Ustilago maydis*)	
9. corneta, cornetilla -fir’s funnel = fir’s little trumpet- (*Turbinellus floccosus)*	
10. cualtzitzi (*Russula delica*)	
11. champiñon (*Agaricus campestris*)	
12. charritos (*Russula delica*)	
13. escobeta -broom- (*Ramaria bonii*, *R*. *cystidiophora*, *R*. *sanguínea*, *R*. *versatilis*)	
14. *gāchupi*, (*Helvella crispa*)	
15. güerito (*Helvella crispa*)	
16. huevitos -little egg- (*Lycoperdon perlatum*)	
17. *ezquilo* (*I*. *gibba*)	
18. negrito -little black- (*Helvella lacunosa*)	
19. olotes (*Morchella elata*, *M*. *esculenta*)	
20. orejas -ear- (*Helvella crispa*)	
21. pante (*Boletus atkinsonii*, *B*. *pinophilus*)	
22. panza -belly- (*Suillus pseudobrevipes*)	
23. popozo, pupuzo, (*S*. *pseudobrevipes*, *Chalciporus piperatus*)	
24. señoritas -miss- (*Hygrophorus chrysodon*)	
25. tamborcito -drum-(*H*. *lacunosa*)	
26. tecajete (*R*. *delica*)	
27. *tecōzah*, tecusa, tecutzal (*C*. *cibarius*)	
28. *tehtecuitl (Armillaria mexicana)*	
29. tlapitzal (*Turbinellus floccosus*)	
30. totomoch (*Infundibulicybe gibba*, *I*. cf. *squamulosa*)	
31. *xelhuāz* --escobeta-broom- (*Ramaria bonii*, *R*. *cystidiophora*, *R*. *rubripermanens*, *R*. *sanguínea*, *R*. *versatilis*)	
32. xitetl =huevitos-egg- (*Lycoperdon perlatum*)	
33. *xocoyoli*, xuxocoyoli, xoxocoyoli *(Laccaria bicolor)*	
34. *xōlētl*, *xulētl* (*L*. *decastes* complex)	
35. *xo*-*tomāh*, *xo*-*tomāhme*, *xo*-*tomāhte*, *xo*-*tomāhtzi*, (*Boletus atkinsonii*, *B*. *pinophilus*)	
Generic taxa designed by productive complex primary names (these names, despite having two constituents, refer to folk genres and refer to a higher taxa)	
1. *cacax*-*nanacatl* (*L*. *indigo*)	
2. *cītlal*-*nanacatl* -hongo de estrellas- star mushroom- (*Amanita muscaria*)	
3. *chichil*-*nacatl* -hongo de chile- chili mushroom- (*L*. *salmonicolor*)	
4. *chil*-*nanacatl* (*L*. *salmonicolor*)	
5. *gachupi*-*nanacatl* (*H*. *crispa)*	
6. *gachupi*-*tzetze* (*H*. *crispa)*	
7. hongo azul -blue mushroom- (*L*. *indigo*)*	
8. hongo de campana -belt mushroom- (*I*. *gibba*, *I*. cf. *squamulosa*)*	
9. hongo de maguey -maguey mushroom- (*Pleurotus opuntiae*)*	
10. hongo de maíz -corn mushroom- (*U*. *maydis*)*	
11. hongo de mata (*L*. *decastes* complex)*	
12. hongo de ocote (*Hebeloma* aff. *mesophaeum*)*	
13. hongo morado -purple mushroom- (*Chroogomphus jamaicensis*)*	
14. *pante*-*nanacatl* (*Boletus atkinsonii*, *B*. *pinophilus*)	
15. pitzu-nanacatl (*A*. *muscaria*, *Amanita* cf. *smithiana*, *Lactarius chelidoniun* var. c*helidonioides*, *Lactarius luculentus*, *Lactarius mexicanus*, *Lactarius* cf. villosus, *Russula* cf. *fragilis*, *Russula grisceacens*, *Russula murrilli* )	
16. xilona-nanacatl, Xilonaltzitzi, Xixilo-nanácatl (*Hygrophorus chrysodon*)	
17. xitetl-nanacatl (*L*. *perlatum*)	
18. xocoyo-nanácatl, Xoxocoyol-nanácatl (*L*. *trichodermophora*)	
Generic taxa designed by unproductive complex primary names	
1. ayotzin, ayutzin. (*Agaricus campestris*)	
2. ayoxóchitl (*A*. *basii*)	
3. cuatecax (*R*. *delica*)	
4. cuatlamanil (*Amanita tuza*)	
5. cuitlacoche (*U*. *maydis*)	
6. chilnanatzi (*L*. *salmonicolor*)	
7. huihuixocatzi (*H*. *crispa*)	
Folk species designed by secondary names	
1. oyametl-chilnanácatl, (*L*. *salmonicolor*)	
2. cuaxua-xoletl (*L*. *decastes* complex)	
3. ocol-xoletl (*L*. *decastes* complex)	
4. oco-xaltoma (*B*. *atkinsonii*, *B*. *pinophilus*)	
5. oyamel-xotoma (*B*. *atkinsonii*, *B*. *pinophilus*)	
6. poposo-rabia (*Ch*. *piperatus*)	
7. tepe-xotoma (*L*. *aurantiacum*)	
8. tlacual-xoletl (*L*. *decastes* complex)	
9. tlapal-tecosa, tlapal-tecosauitl (*Ch*. *jamaicensis* )	
10. tlapal-xotoma (*B*. *atkinsonii*, *B*. *pinophilus*)	
11. tlaxca-xotoma (*B*. *atkinsonii*, *B*. *pinophilus*)	
12. xiteburo (*L*. *perlatum*)	
13. xotoma-rabia (*B*. *miniatopallescens*)	
14. zaca-xotoma (*B*. *atkinsonii*, *B*. *pinophilus*)	

* = Fungi recognized as edible

All the people separated them first into edible and non-edible and later the edible, by the way of preparation to eat. Only a couple of fungi were not recognized as fungi (*Formitopsis pinicola* and *Dacrimyces* sp.). Another pair of fungi was recognized as poisonous (*Boletus luridiformis* and *Amanita muscaria*). In literature, it has been reported that *B. luridiformis* is an edible fungus, but due to the color change due to abuse, most people consider it poisonous.

Finally, a humoral classification is also present in which mushrooms can be designated as either hot or cold due to the effect of consumption. The cold category contains almost all of the mushrooms consumed. It is believed that these can cause stomach ache and gastrointestinal upset if the person eats too much. They therefore have to be prepared with condiments that contrast with their “cold” properties (mainly garlic); hot mushrooms are those that have medicinal benefits including cuitlacoche (*Ustilago maydis*). Of the medicinal mushrooms, 36 species have been used as a remedy by the local healers and 26 are also edibles [38].

A total of 89 names that correspond to 62 fungal species were found during the study of which 65 are of Nahuatl origin and the rest are names used in Spanish (Table 2). A total of 226 species of macromycetes have been identified from the Malinche National Park [39], thus during the San Isidro interviews, the percentage of mushrooms mentioned by participants in this study represent the 27.43% of the local known species. On two different occasions, free listings were obtained, showing that people on average recognised 14.75_(= 20)_ [40] and 7.87_(*n* = 20)_ [41] mushroom names.

**Table 2:** Species of fungi and traditional names in Nahuatl and Spanish, used in San Isidro Buensuceso, Tlaxcala, Mexico

Species and voucher numbers	Nahuatl names	Spanish names
1. *Agaricus campestris* L. AM 1644	*āyoh-tzin* (*āyotl* or *āyutl* = turtle; *āyohtli* = zucchini *tzin* = diminutive reverential, baby turtle or zucchini)	Champiñón
2. *Amanita basii* Guzmán & Ram.-Guill. AM 1598	*āyoh-xōchitl āyotl* = calabaza; *xochitl* = flower) cuazitlal *= ?* *āquiyoxóchitl (quiyotl* or *quiotl =* stem or shoot; *xochitl* = flawer).	flor de calabaza (zucchini blossoms) hongo amarillo (yellow mushroom)
3. *Amanita muscaria* (L.) Lam.* OHT 13	*pitzō-nanacatl* (*pitzōtl* = pig, *nanacatl* = mushroom; pig’s mushroom) *cītlal-nanacatl* (*cītlalli* or *cītlalin*= star, the star fungus, referring to the scales, because they look like stars)	hongo malo o venenoso (poison fungus)
4. *Amanita* cf. *smithiana* Bas* AM 1594	*pitzō-nanacatl* (Ver *A. muscaria*)	hongo malo (poison fungus)
5. *Amanita tuza* Guzmán AM 1747	*cuah-tlamanil, cuhtlal* (c*uaitl* = cabeza; *tlamanil* = ?) *iztāc-nanacatl (iztāc=* white *nanácatl* = mushroom, White mushroom)	-----
6. *Armillaria mexicana* Elías-Román, *et al.* AM 1742	*tehtecuitl* (*tecuitlatl* = slime o *cuitla*, excrement; probably means stone mushroom) *xotlalist* = ? *xocuitlas =* ?	-----
7. *Boletus atkinsonii* Peck AM 1595	*tlacuahuac-xo-tomāh*, *pante-nanacatl* (bread musroom) *tlaxca-xo-tomāh* (tlaxcalli = tortilla; tortilla mushroom)	panza grande (big belly mushroom) pante cemita
8. *Boletus* aff. *edulis* Bull. RCRL 07	*oyametl- xo-tomāh* (*oyametl* = fir) *xo-tomāh, xo-tomāme*, *xo-tomāh*, *xo-tomāhtzi* (icxi = pata, *tomāhuac* = gordo, hongo de pata gorda; o *xitomātl* = tomato, mushroom likes tomato)	panz, pata gorda (fat leg)
9. *Boletus miniato-pallescens* A.H. Sm. & Hesler * AM 1607	*xo-tomāh-rabia* (*xitomātl =* seems to the tomato*; rabia =* means the fungus is poisonous)	hongo malo (poison fungus)
10. *Boletus pinophilus* Pilát & Dermek OHT 05, 22	*xo-tomāh, xo-tomāh-me*, *xo-tomāh-te*, *xo-tomāh-iz* *xitomātl* = mushoom like to the tomato. *tlatlau- xo-tomāh*, *pantenanácatl*, *tlaxca-xo-tomāh*, *zaca-xo-tomāh, tepetl-xo-tomāh* *oco-xal-tomāh* (*ocoxal* = pine litter)	panza grande (big belly mushroom) pante cemita pata gorda (fat leg)
11. *Boletus rubriceps* D. Arora & J.L. Frank RCRL 21	*zaca-xo-tomāh* (*zacatl* = zacate)	panza o pante de zacate (grass belly)
12. *Boletus* sp. 1 Not Number	*tlapal-xo-tomāh*, *tlapal-xo-tomāh* (*tlapalli* = color), *tlatlau-xo-tomāh= ?*	panza de color
13. *Cantharellus complex cibarius* Fr. OHT 25	*tecōzah, tecusah, tecutzal* (*den kuztic* = the yellow; there are a wild yellow flower with the name *teguza*)	amarillitos (yellowish)
14. *Chalciporus piperatus* (Bull.) Bataille * OHT 04	*popozoh-rabia, popuzoh, pupuso* (*popozoh-nallot* = foam, it refers to the appearence of hymenium)	hongo malo (poison fungus)
15. *Chroogomphus jamaicensis* (Murrill) O.K. Mill. *OHT 21*	*tlapal-tecōzah* *tlapal-tecōzah-uitl* (*el tlapalli =* color*,* it refers to a purple *tecōzah*) *xilpatzutl* = ?	hongo morado (purple mushroom)
16. *Hebeloma* aff. *mesophaeum* (Pers.) Quél. AM 1713, RCRL 04	*ocō-xāl-nanacatl* (*ocō-xālli*= pine-litter; mushroom growing in *ocō-xāl*) *rastrojo-nanácatl* (mushroom growing on stubble)	hongo de ocote (ocote mushroom)
17. *Helvella crispa* (Scop.) Fr. OHT 19; AM 1602	*pantalon-nanacatl*, *gachupi* *huihuixocatzi (uiuixqui = débil* *xocatzi = ?*, weak mushroom?) *gāchupitzetze =* ? *soldados-nanácatl* (soldier mushroom) *huevis-nanacatl* (egg mushroom)	*gachupi* blanco (white gachupi) orejas (ears) güerito orejas de ratón (mouse’ ears)
18*. Helvella lacunosa* Afzel. OHT 20, 38	*charro-nanacatl* (charro-mushroom) *cuatlil* (*quaitl* = cabeza, *tlilli* = black, black head)	tamborcito (drum) negrito (bold) *gachupi negro* (black *gachupi*)
19. *Hygrophorus chrysodon* (Bastch) Fr. AK 2831	*chīlona-nanacatl* (*xilotl* = cob *nanacatl* = fungi) *chīlona-ltzitzi (tzitzi = ?*) *chī-chīlona-nanacatl*	señoritas (ladies) blanquitos (little white)
20. *Infundibulicybe gibba* (Pers.) Harmaja. OHT 44, RCRL 05	*ezquilo* (*ezquitl =* scented flower The name refers to the sweet smell of the fungus), *ezquilo* de *oyametl* *totomoch* (*totomachtle =* the peel , corn’s peel)	hongo de campana (bell mushroom) = campanilla
21. *Infundibulicybe* cf*. squamulosa* (Pers.) P. Kumm. OHT 24	*ezquilo de ocotl*, *totomoch* see *I. gibba*	hongo de campana hongo de campana (bell mushroom) = campanilla
22. *Lactarius chelidoniun* var. c*helidonioides* (A.H. Sm.) Hesler & A. H. Sm.* OHT 11	*pitzō-nanacatl* (see *A. muscaria*)	hongo malo (poison fungus)
23. *Lactarius deliciosus* AME 1730	*chīl-nanacatl den ocotl* (*chilli-mushroom, ocotl = ocote= pine*)	-
24*. Lactarius indigo* (Schwein.) Fr. OHT 32	*cācāx-nanacatl* (*cacaxtli = it refers to kind of blue bird*)	hongo azul (blue mushroom)
25. *Lactarius luculentus* Burl.* OHT 09	*pitzō-nanacatl* (see *A. muscaria*)	hongo malo (poison fungus)
26. *Lactarius mexicanus* Kong & Estrada * OHT 07	*pitzō-nanacatl* (see *A. muscaria*) *cuā-te-cax* poison (*cuatlil* = head; *tecaxitl* = stone plate, poisonous mushroom with a head like a stone plate)	hongo malo (poison fungus)
27. *Lactarius salmonicolor* R. Heim *et* Leclair OHT 30, RCRL 02	*chīl-nanacatl* (*chilli-mushroom*) *chilabuelita*, *chimel-nanacatl, chilnanatzi, chichil-nanacatl, oyamelchil-nanacatl*	cajetitos rojos (red cajetitos) trompa de cochino (pig’s trunk)
28. *Lactarius* cf. villosus Clem.* OHT 10	*pitzō-nanacatl* (see *A. muscaria*)	hongo malo (poison fungus)
29. *Laccaria trichodermophora* G.M.M Muell. OHT 18, RCRL 06	*xocoyoli, xuxocoyoli,* *xoxocoyoli, xoxocoyol-nanacatl,* *xocoyol-nanacatl (xogoyolli = the last child in the family, xogoyolti is the plural, xocoyoles =* plural in Spanish. The name is given because it is one of the smallest edible mushrooms)	Clavito (little nail)
30. *Leccinum aurantiacum* (Bull.) Gray * AM 1606	*tepe-xo-tomāh* (*tepetomatl* = árbol madroño. It is one *xo-tomāh* growing around arbutus (*Arbutus* spp.)	hongo malo (poison fungus)
31. *Lycoperdon perlatum* Pers. AM 1615, OHT 14	*xiteburo*, *xite-nanacatl*, *xitetl* (*xiuitl* = red grass; tetl = huevo; buro = the simplification of the word donkey; donkey egg mushroom) *cefamil* = ?	huevitos (little eggs)
32. *Lycoperdon* sp. AM 1605	xiteburo, xite-nanacatl, xitetl *cefamil* see *L. perlatum*	huevitos (little eggs)
33. *Lyophyllum* sp. 1. AM 1741	*xōlētl, xulētl (xōlētl* = delicate, *xolectle* fragil, *xoleme* es el plural) *ocol-xōlētl* (*ocotl* = ocote)	clavitos (little nails) hongo de mata (caespitose mushroom)
34. *Lyophyllum* sp. 2 AM, 1625, RCRL 09	*tlacual-xōlētl =* (*tlacuahuac* = encino), *cuaxua-xōlētl* = ?	-
35. *Lyophyllum* sp. 3 AM 1764	*oyametl-xōlētl*	-
36. *Morchella elata* Fr. OHT 31	*ōlō-nanacatl(ōlōtl*= corncob. The name is for several species of *Morchella*)	olotes (corncobs)
37. *Morchella esculenta* (L.) Pers. AM 857	*ōlō-nanacatl* see *M. elata.*	olotes (corncobs)
38. *Paneolus* sp. S/N	*nanacatl den kuitlatl*	Hongo de estiércol
39. *Pleurotus opuntiae* (Durieu et Lév.) Sacc. AM 974	*me-nanacatl (metl* = maguey *nanacatl* = mushroom) *meso-nanacatl (*megotl = maguey mushroom) *huexo-nanacatl (huexotl* = willow, willow musroom)	hongo de maguey (maguey mushroom)
40. *Phaeoclavulina abietina* (Pers.) Glachini * AM 1600	*xelhuas den pitzō-nanacatl* (*xelhuas* = broom, poisonous broom)	escobeta venenosa (poison broom)
41. *Phaeolus shweinitzii* (Fries.) Pat. S/N	*nanacatl den kuahuitl*	hongo de tronco
42. *Ramaria apiculata* (Fr.) Donk * E-T 2304	*xelhuas* den *pitzō-nanacatl* (see *R. abietina*)	escobeta venenosa (poison broom)
43. *Ramaria bonii* Estrada AM 1599	*xelhuāz* (*xelhuāztle =* broom, mushroom’ broom)= *x. den kustic*	escobeta amarilla (broom)
44. *Ramaria concolor* (Corner) R. H. Petersen* AM 1601	*xelhuāz den pitzō-nanacatl*	hongo malo (poison fungus)
45. *Ramaria cystidiophora* (Kauffman) Corner AM 1715D	*xelhuāz* (see *R. bonii)*	Escobeta (broom)
46. *Ramaria rubricarnata* (*Pers.*) *ersatilis* Quél. AM 1762	*xelhuāz* (see *R. bonni*).	Escobeta rosa (broom)
47. *Ramaria rubripermanens* Marr & D.E.Stuntz AM 1715C, 1747A	*xelhuāz* (see *R.* bonii) *cuamanox = ?* *xelhuāz-tzitzi* (tzitzi = ?)	escobeta morada (purple broom) escobeta café
48. *Ramaria sanguinea* (Pers.) Quél. AM 1747B	*xelhuāz* (see *R*. bonii)	Escobeta (broom)
49. *Ramaria* sp.1 Subgenus *laeticolora* AM 1681	*xelhuāz* café	escobeta café
50. *Ramaria* sp. 2 RCRL 57	*cuamanox*	-
51. *Russula delica* group OHT 15, RCRL 01	*cuā-te-cax* (*cuatlil = cabeza, tecaxitl =* stone plate or metate) *cualtzitzi (cuatlil =* head*, tzitzi =* ?) *iztāc nanacatl* (*iztāc =* white *nanacatl* = mushroom)	charritos tecajete
52. *Russula* cf*. fragilis* Vittad.*AK 2924	*pitzō-nanacatl*	hongo malo (poison fungus)
53. *Russula griseascens* (Bon & Gaugué) Marti* AK 2939	*pitzō-nanacatl*	hongo malo (poison fungus)
54. *Russula murrilli* Burl.* AM 1613	*pitzō-nanacatl*	hongo malo (poison fungus)
55. *Sarcodon* sp. * S/N	*tlalpīltzal den pitzō-nanacatl*	corneta de veneno (poison trumpet)
56. *Suillus pseudobrevipes* A.H. Sm. *et* Thiers AM 1596	*popozo, pupuzo* (*popozonallot* = foam; foam mushroom)	panza (belly mushroom) pancita chica (small bely)
57. *Trametes* sp. S/N	*nanacatl den kuahuitl*	Hongo de tronco
58. *Tricholoma equestre* (L.) P. (Kumm) AET 2306	*-*	cailita (?)
59. *Turbinellus floccosus* (Schwein.) Earle ex Giachini & Castellano AM 1609	*tlalpīltzal* (*tlalpīltzalli=* trumpet, trumpet shaped mushroom) *oyametl-nanácatl* (*oyametl* = fir, *nanacatl* = mushroom, fir mushroom) = *oyametl-tlapiltzal*	corneta (trumpet = funnel mushroom) cornetilla (trumpet = funnel mushroom)
60. *Tylopilus* sp. AM 2017A	*tepeto-- xo-tomāh*, *tepetl = cerro*	panza del cerro
61. *Ustilago maydis* (DC.) Corda AM 973	*cuitlacoche* (*cuitla* = excrement *cochi =* pig; pig’s excrement)	hongo de maíz (corn’s mushroom) cochinito (little pig)
62. *Xerocomus truncatus* (Singer, Snell & E.A. Dick)* OHT 03	*xo-tomāh-rabia* (It is a poisonous *xo-tomāh*)	hongo malo (poison fungus)

* = Names including species considered poisonous.

### Determination of the criteria used in the SIBS classification system

The SIBS pragmatic classification is based on the distinction between edibles and non-edibles and therefore meets the utilitarian criteria. However, it also uses morphological characteristics so the system is a mixed one. Thus the groupings made based on gastronomical criteria seem reasonable as it also evidences a knowledge of the intrinsic properties of the mushrooms (e.g., textures, consistency, and structural morphology). Further, the resulting groupings are a demonstration of a larger knowledge mosaic of perceptual characteristics of these organisms, as well as phenological and ecological characteristics that enable the people from SIBS to discriminate between the two main groups of mushrooms recognized mushrooms (edibles and inedibles). In a similar way, Lira-Franco [31] founded that in the locality of Tepulco, Puebla (on the outskirts of La Malinche volcano but in Puebla state), people use many characteristics to separate the edible mushrooms from the inedible and poisonous ones, like the specific phenology of the species or their habitats.

The population of SIBS can be described as an ethnic group in transition meaning that acculturation and urbanization influence the amount of time they spend in contact with nature and this can reduce the reliability of ethnobiological information within the community [9, 42].

Campos-Rivera [43] pointed out that ethnobotanical knowledge among the SIBS population stabilizes at 16 years. This is higher than that reported elsewhere, being a possible indicator of knowledge loss, along with other factors such as the change in economic activities, the migration to cities, school attendance, and the decline of the Nahuatl language among young people. As for adults, knowledge is more homogeneous, and varies mainly in relation to linguistic competence (whether they speak Nahuatl or not). This indicates the importance of language in maintaining knowledge of plants and it is not possible to disassociate the conservation and use of resources from the cultural characteristics of a society.

The classification of fungi in SIBS reflects the nature of these organisms and their seasonal variability and the difference in fructification between species. Mushrooms tend to be restricted to the rainy season causing limited and inconsistent availability of the resource, unlike plants or animals, with a few exceptions such as cultivated mushrooms like *Agaricus bisporus* and *Pleurotus* spp.). Furthermore, not every mushroom necessarily occurs in every season nor in the same month with changes occurring from one rainy season to another (year to year). Another characteristic of these organisms is the variation in their habitat which is related to the associations they form with plants or specific substrates, along with climatic factors and their own genes. Any of these characteristics could be controlling the contact communities have with mushrooms affecting the knowledge that some people may have of these organisms. However, the people of SIBS use mushrooms as an important resource for alimentary and economic subsistence during the rainy season and local specialists in mushroom foraging continue to collect throughout the year.

## Discussion

Berlin et al. [6] consider that groupings made using utilitarian criteria are not valid as folk taxonomy; nevertheless, it is observed that in other studies on the topic such as those by Alcorn [44], Morris [15], Turner [45, 46], Johnson and Hargus [47], and Johnson [48], perceptual and functional criteria coexist inside a single classification system in which are found groups with nameless members due to their lack of utility. Investigations done in Mexico show that utilitarian criterion is used in conjunction with other criteria such as morphological and ecological attributes; this has been reported in studies by Mapes et al. [7], Chacon [49], Estrada-Torres and Aroche [27], Gispert et al. [9], and Mata [10].

Chacon [49] evidenced a fairly simple division that separates these organisms according to the substrate of growth, as in SIBS, in addition to the one obtained based on edibility criteria. It is important to recognize at this point, the work of Aniceto-Crisostomo [8] since it has some similarities with the SIBS study. It observed that the classification of mushrooms follows strictly utilitarian criteria and that inhabitants divide mushrooms into three groups: edible, those not eaten because they are poisonous (species here have a name), and the ones that are not eaten because they are unknown and remain unnamed.

Different criteria make possible the occurrence of different ways in which to classify a resource or group of organisms within the same area as demonstrated in the study of Palomino-Naranjo [11] which presents three classifications used by the inhabitants of San Juan Atzingo, State of Mexico (ecological, phenological and utilitarian). This can cause confusion in trying to find a unified and unique classification system that is applicable to the group in question. This situation was also presented in SIBS.

### Nomenclature analysis and proposed classification system for fungi from SIBS

**Fig 6:**
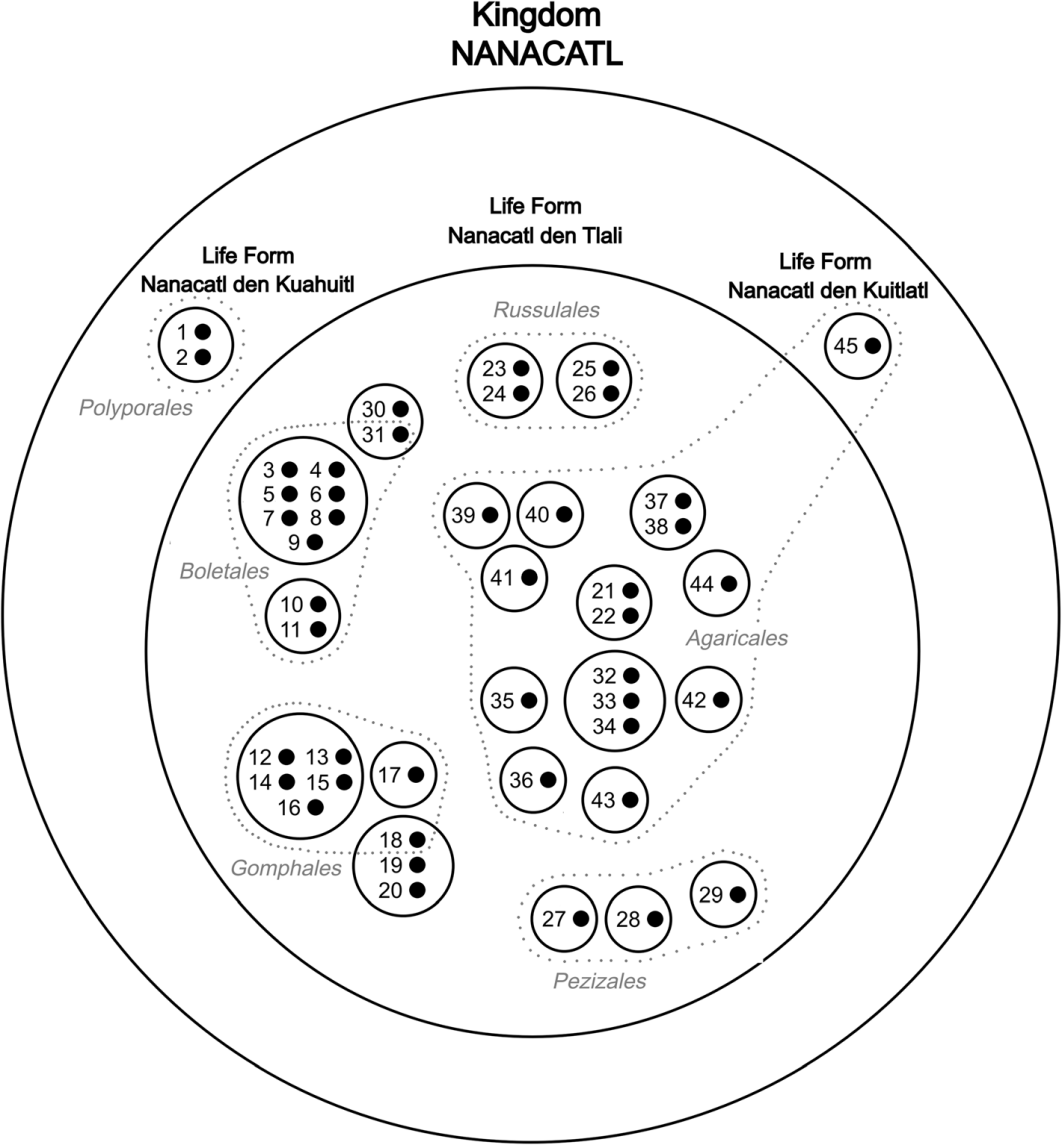
Schematic representation of the nahua classification of fungi in San Isidro Buensuceso, Central Mexico. Numbers with a black dot to the right represent biological species: Small black circles with one to seven biological species inside represent ethnogeneric taxa. The three main life forms are marked with their Nahuatl names. Gray dotted lines and names represent the biological orders of the Fungi. Biological species with their names in Nahuatl: 1. Nanacatl den kuahuitl (*Trametes* sp.), 2. Nanacatl den kuahuitl (*Phaeolus schweinitzii*), 3. Oyametl-xo-tomāh (*Boletus* aff. *edulis*), 4. Tlacuahuac-xo-tomāh (*B*. aff. *atkinsonii*), 5. Oco-xo-tomāh (*B*. *pinophilus*), 6. Zaca-xo-tomāh (*B*. *rubriceps*), 7. Tepetl-xo-tomāh (*Tylopilus* sp.), 8. Tlapal-xo-tomāh (*Boletus* sp. 1), 9. Xo-tomāh-rabia (*Boletus miniato*-*pallescens*), 10. *Popozoh*-*rabia* (*Chalciporus piperatus*), 11. Popozoh-nanacatl (*Suillus pseudobrevipes*), 12. Xelhuāz-nanacatl den kustic (*Ramaria bonii*), 13. Xelhuāz-nanacatl den cafe *(Ramaria* sp.1), 14. Xelhuāz-nanacatl den morada (*R*. *rubripermanens*), 15. Xelhuāz-nanacatl rosa (*R*. *rubricarnata*), 16. Xelhuāz-nanacatl den pitzō-nanacatl (*R*. *apiculata*), 17. Cuamanox (*Ramaria* sp. 2), 18. Oyametl-tlapiltzal *(Turbinellus floccosus*), 19. Tlalpīltzal den pitzō-nanacatl *(Sarcodon* sp.), 20. Tlalpīltzal den blanco (?), 21. Ezquilo den oyametl (*Infundibulicybe gibba*), 22. Ezquilo den ocotl (*I*. cf. *squamulosa*), 23. *Cuā*-*te*-*cax* (*R*. *delica* group), 24. Cuā-te-cax azul (*Lactarius indigo*), 25. Oyamelchil-nanacatl (*L*. *salmonicolor*), 26. Chil-nanacatl den ocotl (*L*. *deliciosus*), 27. Gachupi-nanacatl (*Helvella crispa*), 28. Charro-nanacatl (*H*. *lacunosa*), 29. Ōlō-nanacat (*Morchella* sp.), 30. Tecōzah den kustic (*Cantharellus* aff. *cibarius*), 31. Tlapal-tecōzah-uitl (*Chroogomphus jamaicensis*), 32. Ocotl-xōlētl (*Lyophyllum* sp. 1), 33. Tlacual-xōlētl (*Lyophyllum* sp. 2), 34. Oyametl-xōlētl (*Lyophyllum* sp. 3), 35. Xocoyol-nanacatl (*Laccaria trichodermophora*), 36. Chīlona-nanacatl (*Hygrophorus chrysodon*), 37. Xite-nanacatl (*Lycoperdon perlatum*), 38. Cefamil (*Lycoperdon* sp.), 39. Cītlal-nanacatl (*Amanita muscaria*), 40. Āyoh-xōchitl (*A*. *basii*), 41. Iztāc-nanacatl (*A*. *tuza*), 42. Tehtecuitl (*Armillaria mexicana*), 43. Me-nanacatl (*Pleurotus opuntiae*), 44. Āyoh-tzin (*Agaricus campestris*), and 45. Nanacatl den kuitlatl (*Panaeolus* sp.)

Analysis of the nomenclature suggests that the taxonomic structure used in SIBS includes four levels or categories: kingdom (unique beginner), life form, generic, and specific. Berlin [6] recognized that taxa from traditional classification systems are distributed between four to six ethnobiological ranks. The hierarchical structure that is present in SIBS satisfies such an approach hence fulfilling the required characteristics to affirm the existence of a minimum accepted folk classification. The graphic representation of the Nahua classification of fungi can be seen in Fig. 6.

#### Kingdom level

The word nanacatl which is used in SIBS to refer to mushrooms represents the taxa of the level kingdom. Berlin [6] noted that on some occasions, the taxon kingdom (level 0, unique beginner) receives a specific name or is tagged by a primary lexeme. This is precise and defines the taxon kingdom, separating it from other organisms such as animals (yolkatl) and plants (xihuitl). According to Martín del Campo [32], the word nanacatl means meat. This is analogous to the lexeme nyama which means wild animal or meat. It is used by the Chewas from Malawi to name edible mushrooms because the flavor and texture of such are more like those of animal meat than those of plants. Morris [15] concluded that plants and mushrooms were different categories in the Chewa cosmovision and is probably further evidence of the conceptual separation between plants and mushrooms in the Mesoamerican region. Evidence from other parts of the world shows the use of an ethnobiological category for mushrooms labeled by a lexeme, showing the most inclusive hierarchy in the domain boundaries as well as degree of lexication [50]. Taylor [51] reports that Tobelo, on outskirts of Halmahera, Indonesia, have a category o gauku which includes mushrooms and shelf fungi, also the pinatubo negrito people from Philipines use the term kwat for terrestrial mushrooms, and the peasants from La Paz, in Córdova Argentina, use the word hongo. People belonging to original groups from Mexico also use a certain term to group fungi; terekuicha by the Purepecha from Michoacan [7]; cikinte from Huastec [52]; canul te’tik by the Tzotzil of Zinacantan, Chiapas [53]; “*ccho*” for the Matlazincas from Nevado de Toluca [54]; thain in the Mazatec of Oaxaca [3]; nanagame among the Nahuas of Hueyepan, Morelos [55]; chejchew by the Tzeltal from Chiapas [13]; kuxum by the Lacandon from Naha, of the same state although this term is not exclusively used for macro fungi being also applied to micro fungi such as molds [56]; tonkgolo is the name for fungi in the Tutunakú language of Zongozotla, Puebla [57]; Jo en la lengua ñuhmu de Ixtenco, Tlaxcala [58] y kho en la región de Acambay, Estado de México [27].

Other characteristics indicated by Berlin [6] are observed in the classification system of SIBS. Vocabulary is well developed and linked specifically to the organisms in this category thus there are specific linguistic markers (e.g., the word nanacatl) that indicate that the organisms are a part of this category and not of others (like animals and plants). This shows that they are a distinct group. There are specific terms employed to refer to this group of organisms and some fungal basidiomata, thus demonstrating that there are specific terms for the anatomical structure of the mushroom. In a similar way that was indicated by Lampman [13] for the Tzetal from Chiapas, specific terms to describe morphological characters exist and as such depict many attributes such as size, color, place, and season of fructification, odor, taste, and in SIBS, the association with plants, interaction with animals, and the ways it can be prepared or cooked for its consumption.

#### Life form taxa

**Fig. 7:**
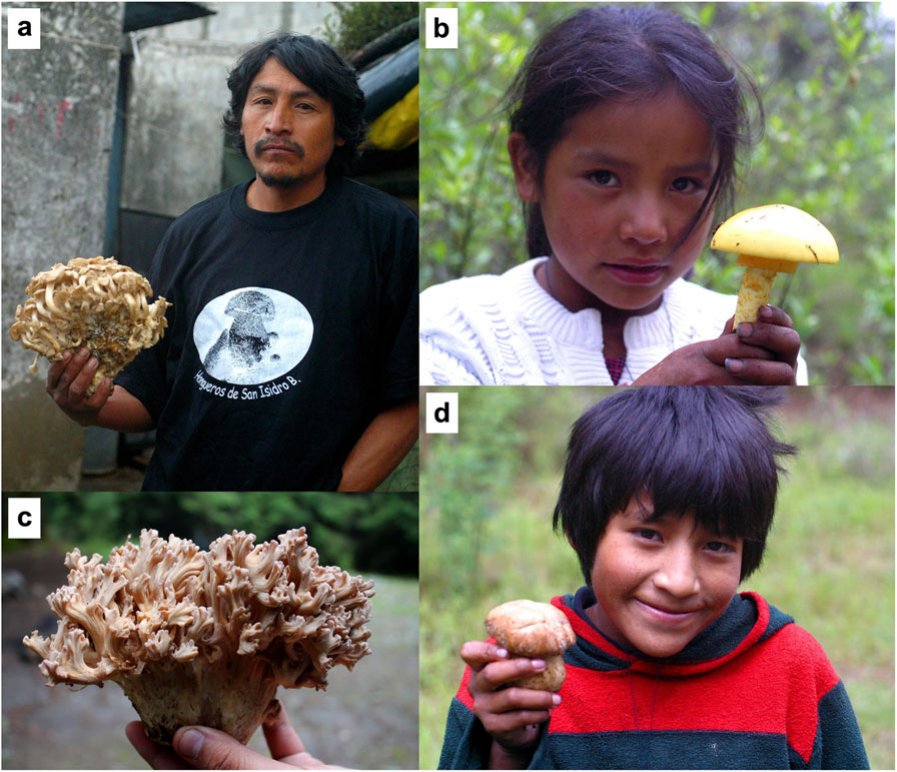
Examples of different fungi species which are in the taxa nanacatl den tlali, considered as cualinanacatl by Nahua people from San Isidro Buensuceso, Mexico. **a** Domingo Domínguez holding a xuletl (*Lyophyllum decastes* complex). **b** Adriana Mariel with an āyoh-xōchitl (*Amanita basii*). **c** xelhuāz-nanacatl (*Ramaria* sp.). **d** A boy (Miguel Domínguez) with a young fruiting body of xo-tomāh (*Boletus pinophilus*)

The pile sorting exercises carried out in this study showed that people in SIBS divide mushrooms into two groups: nanacatl den tlali (mushrooms that grow on the ground) and nanacatl den cuahuitl (mushrooms that grow on wood), although the term estiercol-nanacatl (mushrooms that grow on dung) was also mentioned during the course of field work with the community. Berlin [6] pointed out that the life form category usually includes between five and ten members, especially in the case of plants and animals. In this case, this category is poorly represented having only three members. Life form taxa include the majority of lower range taxa (generic and specific). The same author states that, linguistically, life form taxa are depicted by primary lexemes (e.g., tree or mammal). In SIBS, the name comprises the word applied for the immediate superior category (nanacatl) plus a word that references the substrate on which the associated mushrooms grow (e.g., nanacatl + den tlali). It is proposed that these categories may correspond to the life form taxon. Nevertheless, it is necessary to conduct a more profound linguist research programme to determine the existence of more members, if they exist, and their tangibility. These three groups denote a division made by the function of the substrate in which the mushrooms grow. Some examples of the highest important mushrooms in this category are in Fig. 7.

#### Generic taxa

We recognized a total of 24 taxa generic folk in the Nahuatl categorization of fungi. Spanish and Nahuatl names are used by people to name these taxa, some of them are synonyms. The names corresponding to the generic taxa are designated by simple primary lexemes which may or may not denote membership in a higher level category (meaning that they include a word indicating that they belong to a more inclusive category). The vast number of names tagged by productive primary lexemes denotes a hierarchical structure by including in such names the word nanacatl which refers to the main category that depicts the large group of known fungi in SIBS. The generic category is constituted by primary lexemes, namely root words and simple or compound words that may or may not denote belonging to a certain immediate superior category (i.e., the name may or may not include the term that labels the great category of mushrooms, e.g., ezquilo is the name given to *Infundibulicybe gibba* and it can be called only as such or it can also include the nanacatl (forming the name ezquilo-nanacatl).

There are certain (scientific) species that have assigned variations of the same Nahuatl name for example: ezquilo, ezquilo-nanacatl, ezquilon (*I*. *gibba*); gachupi-nanacatl, gachupi (*Helvella crispa*); chīl-nanacatl, oyamel-chīl-nanacatl (*Lactarius salmonicolor*).

Out of the total number of names (including Nahuatl and Spanish) (89), 39.33% (35) are generic names designed by simple primary lexemes, 20.22% (18) are generic names designated by productive primary lexemes, 16.9% (7) are generic names designated by unproductive primary lexemes, and 19.1% (14) are names of species designated by secondary lexemes (Table 3).

**Table 3:** Categorization of traditional names (in Nahuatl and Spanish) for fungi used in SIBS, Mexico according to Berlin et al. [6]

Generic taxa designed by simple primary names	
1. amarillitos -little yellow- (*Cantharellus cibarius*)	
2. blanquitos -little white- (*Hygrophorus chrysodon)*	
3. cailita ? (*Tricholoma flavovirens*)	
4. campanilla -beltl- (*Infundibulicybe gibba*, *I*. cf. *squamulosa*)	
5. cefamil ? (*Lycoperdon perlatum*)	
6. cemita .-a kind of bread- (*Boletus atkinsonii*, *Boletus pinophilus*)	
7. clavitos -nails- (*Lyophyllum decastes* complex)	
8. cochinito -little pig- (*Ustilago maydis*)	
9. corneta, cornetilla -fir’s funnel = fir’s little trumpet- (*Turbinellus floccosus)*	
10. cualtzitzi (*Russula delica* group)	
11. champiñon (*Agaricus campestris*)	
12. charritos (*Russula delica*)	
13. escobeta -broom- (*Ramaria bonii*, *R*. *cystidiophora*, *R*. *sanguínea*, *R*. *versatilis*)	
14. *gāchupi*, (*Helvella crispa*)	
15. güerito (*Helvella crispa*)	
16. huevitos -little egg- (*Lycoperdon perlatum*)	
17. ezquilo (*I*. *gibba*)	
18. negrito -little black- (*Helvella lacunosa*)	
19. olotes (*Morchella elata*, *M*. *esculenta*)	
20. orejas -ear- (*Helvella crispa*)	
21. pante (*Boletus atkinsonii*, *B*. *pinophilus*)	
22. panza -belly- (*Suillus pseudobrevipes*)	
23. popozo, pupuzo, (*S*. *pseudobrevipes*, *Chalciporus piperatus*)	
24. señoritas -miss- (*Hygrophorus chrysodon*)	
25. tamborcito -drum-(*H*. *lacunosa*)	
26. tecajete (*R*. *delica* group)	
27. *tecōzah*, tecusa, tecutzal (*C*. *cibarius*)	
28. *tehtecuitl (Armillaria mexicana)*	
29. tlapitzal (*Turbinellus floccosus*)	
30. totomoch (*Infundibulicybe gibba*, *I*. cf. *squamulosa*)	
31. *xelhuāz* --escobeta-broom- (*Ramaria bonii*, *R*. *cystidiophora*, *R*. *rubripermanens*, *R*. *sanguínea*, *R*. *versatilis*)	
32. xitetl =huevitos-egg- (*Lycoperdon perlatum*)	
33. *xocoyoli*, xuxocoyoli, xoxocoyoli *(Laccaria bicolor)*	
34. *xōlētl*, *xulētl* (*L*. *decastes* complex)	
35. *xo*-*tomāh*, *xo*-*tomāhme*, *xo*-*tomāhte*, *xo*-*tomāhtzi*, (*Boletus atkinsonii*, *B*. *pinophilus*)	
Generic taxa designed by productive complex primary names (these names, despite having two constituents, refer to folk genres and refer to a higher taxa)	
1. *cacax*-*nanacatl* (*L*. *indigo*)	
2. *cītlal*-*nanacatl* -hongo de estrellas- star mushroom- (*Amanita muscaria*)	
3. *chichil*-*nacatl* -hongo de chile- chili mushroom- (*L*. *salmonicolor*)	
4. *chil*-*nanacatl* (*L*. *salmonicolor*)	
5. *gachupi*-*nanacatl* (*H*. *crispa)*	
6. *gachupi*-*tzetze* (*H*. *crispa)*	
7. hongo azul -blue mushroom- (*L*. *indigo*)*	
8. hongo de campana -belt mushroom- (*I*. *gibba*, *I*. cf. *squamulosa*)*	
9. hongo de maguey -maguey mushroom- (*Pleurotus opuntiae*)*	
10. hongo de maíz -corn mushroom- (*U*. *maydis*)*	
11. hongo de mata (*L*. *decastes* complex)*	
12. hongo de ocote (*Hebeloma* aff. *mesophaeum*)*	
13. hongo morado -purple mushroom- (*Chroogomphus jamaicensis*)*	
14. *pante*-*nanacatl* (*Boletus atkinsonii*, *B*. *pinophilus*)	
15. pitzu-nanacatl (*A*. *muscaria*, *Amanita* cf. *smithiana*, *Lactarius chelidoniun* var. c*helidonioides*, *Lactarius luculentus*, *Lactarius mexicanus*, *Lactarius* cf. villosus, *Russula* cf. *fragilis*, *Russula grisceacens*, *Russula murrilli* )	
16. xilona-nanacatl, xilonaltzitzi, xixilo-nanácatl (*Hygrophorus chrysodon*)	
17. xitetl-nanacatl (*L*. *perlatum*)	
18. xocoyo-nanácatl, xoxocoyol-nanácatl (*L*. *trichodermophora*)	
Generic taxa designed by unproductive complex primary names	
1. ayotzin, ayutzin. (*Agaricus campestris*)	
2. ayoxóchitl (*A*. *basii*) 3. cuatecax (*R*. *delica* group) 4. cuatlamanil (*Amanita tuza*) 5. cuitlacoche (*U*. *maydis*) 6. chilnanatzi (*L*. *salmonicolor*) 7. huihuixocatzi (*H*. *crispa*)	
Folk species designed by secondary names	
1. oyametl-chilnanácatl, (*L*. *salmonicolor*)	
2. cuaxua-xoletl (*L*. *decastes* complex)	
3. ocol-xoletl (*L*. *decastes* complex)	
4. oco-xaltoma (*B*. *atkinsonii*, *B*. *pinophilus*)	
5. oyamel-xotoma (*B*. *atkinsonii*, *B*. *pinophilus*)	
6. poposo-rabia (*Ch*. *piperatus*)	
7. tepe-xotoma (*L*. *aurantiacum*)	
8. tlacual-xoletl (*L*. *decastes* complex)	
9. tlapal-tecosa, tlapal-tecosauitl (*Ch*. *jamaicensis* )	
10. tlapal-xotoma (*B*. *atkinsonii*, *B*. *pinophilus*)	
11. tlaxca-xotoma (*B*. *atkinsonii*, *B*. *pinophilus*)	
12. xiteburo (*L*. *perlatum*)	
13. xotoma-rabia (*B*. *miniatopallescens*)	
14. zaca-xotoma (*B*. *atkinsonii*, *B*. *pinophilus*)	

* Fungi recognized as edible

Specific and varietal taxa are separated from others by few characteristics and exhibit a hierarchical arrangement with the generic names [6]. This can be exemplified by the following genera like xōlētl (*Lyophyllum decastes*) and xō-tomah (*Boletus atkinsonii*, *B*. *pinophilus*), with the exception of tepe-xō-tomah (*Leccinum aurantiacum*) which corresponds to a fungus considered to be poisonous:

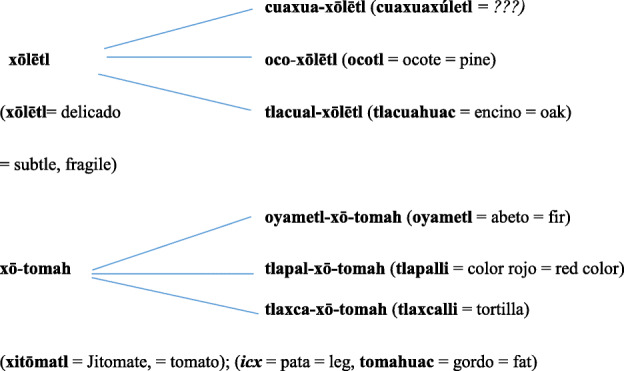



#### Specific taxa

The number of folk-specific taxa for wild fungi included 30 etnotaxones. They were in higher proportion than generic taxa. It is different from the statement done by Berlin [6] who indicated that most numerous taxa in folk biological taxonomies will be taxa of generic rank, and the 80% of folk generic are monotypic and include no taxa of lesser rank. The author also said that there is some evidence to suggest that the recognition of subgeneric taxa appears to be motivated in part by cultural considerations. The latter corresponds partially with the folk species related to the folk genera xōlētl and xō-tomah that result from culinary interest for the people of SIBS and also have economic relevance.

Berlin [6] pointed out that possibly foraging societies have poorly developed or lack entirely taxa of specific rank. It will be occurring in SIBS, people are changing foraging activities because they go outside the town to the big city to get a “better job.”

Under the present classification, 14 names with secondary lexemes were found. The secondary lexemes modify the generic name through an attribute and correspond to specific taxa. The varietal taxa are not represented due to the lack of linguistic evidence that would support their presence in the names of mushrooms used by the inhabitants of SIBS.

**Fig. 8:**
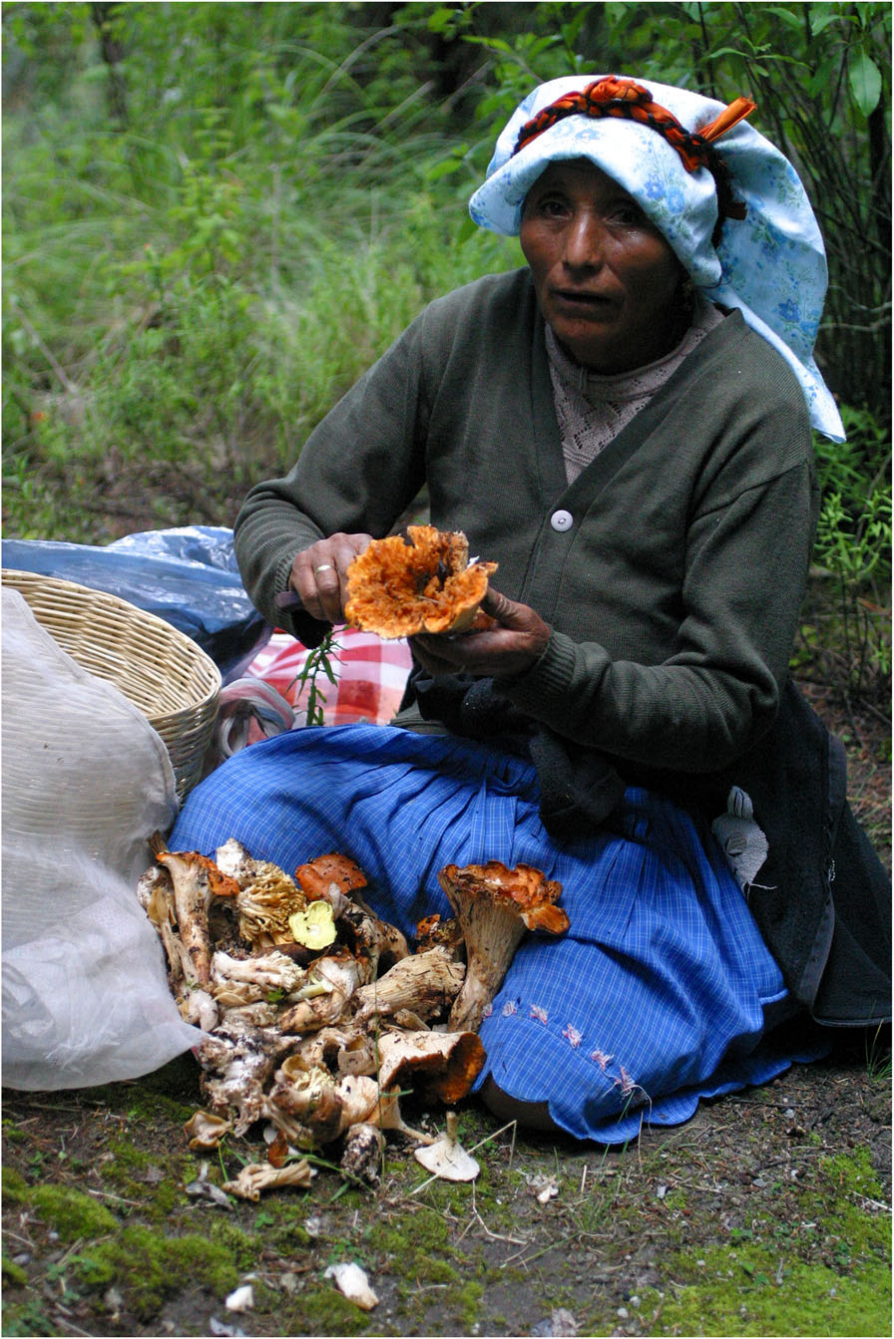
Lady (Herlinda Arcce) from San Isidro cleaning some tlapitzal (*Turbinellus floccosus*), the species with highest cultural significance as food. This fungus is identified because of its trumpet shape; it has a special cleaning to be eaten

The criteria used for the recognition of fungi are very important to ensure the precise identification of edible species. In this sense, the Nahuas of SIBS have a fairly reliable knowledge, based on the experience of observing, collecting, and consuming these organisms over many decades. Fungi are identified by collectors through the observation of a set of characteristics that become latent in people’s minds at the time of the observation of a species. These include knowledge of the precise sites in which they bear fruit, the associations with particular tree species, the general morphology that includes the colors, the color changes, the presence or absence of particular structures, and the different odors that characterize edible species or those considered toxic (Fig. 8). Many times when asked how do you know that this fungus is edible? They respond: “I already know it.” That answer implies that in the presence of a fungus, all the accumulated knowledge is activated through excessive daily practice in the rainy seasons. The information obtained is a cultural and biological representation of a group that shows the way in which this unit is concentrated in the language, which at one time could be more homogeneous and extended, but which over time changes with different social circumstances. Through the analysis of the names and the way in which they group the fungi, the close relationship between the Nahuas of SIBS and the wild fungi is evident.

Likewise, the grouping of fungi based on the forms of preparation provide evidence of the observation of criteria such as the consistency of the fruiting bodies, the texture, and the flavor, which are all characteristics of great importance for the preparation of food.

## Conclusion

With the methods used in this research, the concept of fungi as a distinctly different group from plants, animals, and food changed in relation to the stimuli that were used in the pilot tests. Our findings suggest that fungi are considered to be a food but were also grouped as a distinct ensemble from animals and plants. The results obtained give evidence that the mushrooms are recognized by the SIBS Nahuas as a kingdom, with three forms of life (land fungi, wood fungi, and manure fungi) unlike the Zapotecs from San Pedro and San Juan Mixtepec, Oaxaca.

Fungi are not considered a form of life for the Nahuas of SIBS, in the same way that they are for the Zapotecs from San Pedro and San Juan Mixtepec, Oaxaca [5] since in this case, it was shown that nanacatl is divided into three groups depending on the substrate in which they develop. From an academic point of view, unlike those that occur in plants, fungi lifestyles are recognized and defined based on the way in which they obtain their food: saprotrophs, parasites, or mutualists. Whereas we found in SIBS, that life forms are defined according to the substrata which is an ecological strategy.

During the course of our research, whether related to Mexico or in other parts of the world, it has been observed that the fungal traditional folk schemes that have been proposed are the result of different sources of evidence, the linguistic, pragmatic, or ecological. The analysis of the fungi names in SIBS shows the similarity in relationships that have a hierarchical structure. So is it possible that the evolution of societies over time is causing these changes in the approach to way of ordering or grouping nature? We have no information about historic fungal studies but language is an example that gives us a lot of information. It remains to do more studies on this topic locally and also in other parts of Mexico and the world to gain more evidenced conclusions in this regard. It is also clear that each human group and even each person has different ways of relating to nature and this is expressed in the classification and nomenclature.

It is also necessary to conduct more detailed research with a wider audience using lessons learnt from the methodology applied in this preliminary study in order to provide more detailed evidence to support the preliminary findings on the traditional folk classification system for mushrooms used by the SIBS. The use of the multi-dimensional model suggested by Alcántara-Salinas et al. [59] would be a very interesting and alternative approach and could either reinforce the information obtained or show that another way of processing the information may generate another outcome.

## Data Availability

Not applicable.

## References

[1] Moreno-Fuentes A, Garibay-Orijel R, Tovar-Velzco J, Cifuentes J (2001). Situación actual de la etnomicología en México y el mundo. Etnobiología..

[2] Garibay-Orijel R (2000). *La etnomicología en el mundo: pasadp*, *presente y futuro*.

[3] Wasson RG (1983). El hongo maravilloso: Teonanácatl.

[4] Moreno-Fuentes A, Garibay-Orijel R. *La Etnomicología en México: una introducción al estado del arte*. Red de Etnomicología y Patrimonio biocultural (CONACyT)-Universidad Autónoma del estado de Hidalgo-Instituto de Biología (UNAM)-Sociedad Mexicana de Micología-Asocoación Etnobiológica Mexicana, A.C., Grupo Interdisciplinario para el Desarrollo de la Etnomicología en México-Sociedad Latinoamericana de Etnobiología, México City; 2014.

[5] Hunn ES, Venegas-Ramírez Y, Vázquez Dávila MA. Where do fungi fit? The fungal domain in Mixtepec Zapotec. *J*.*Ethnobiol*. 2015;35(2):286-313. https://doi.org/10.2993/etbi-35-02-286-313.1.

[6] Berlín B (1992). Ethnobiological classification: principles of categorization of plants and animals in traditional societies.

[7] Mapes C, Guzmán G, Caballero J. 1981. *Etnomicología purépecha*. *El conocimiento y uso de los hongos en la Cuenca de Pátzcuaro*, *Michoacán*. México: Serie etnociencia 2. Dirección General de Culturas Populares, Secretaría de Educación Pública, Sociedad Mexicana de Micología AC; 1981.

[8] Aniceto-Crisóstomo E (1982). *Los hongos en la región mazahua*. *Unidad Regional Pátzcuaro*, *Michoacán*.

[9] Gispert M, Nava O, Cifuentes J (1984). Estudio comparativo del saber popular de los hongos en dos comunidades de la sierra del Ajusco. Bol Soc Mex Mic..

[10] Mata G (1987). Introducción a la etnomicología maya de Yucatán. El conocimiento de los hongos de Pixoy, Valladolid. Rev Mex Mic.

[11] Palomino-Naranjo A (1992). Etnomicología tlahuica de San Juan Atzingo, Estado de México.

[12] Rúan-Soto F, Mariaca-Méndez R, Cifuentes J, Limón-Aguirre F, Pérez-Ramírez L, Sierra-Galván S (2005). Etnobiología.

[13] Lampman AM (2007). General principles of ethnobiological classification among the Tzeltal Maya of Chiapas, México. J Ethnobiol.

[14] Morris B (1984). Macrofungi of Malawi: some ethnobotanical notes. Bull Brit Myc Soc.

[15] Morris B (1984). The pragmatics of folk classification. J Ethnobiol.

[16] Morris B (1987). The folk classification of fungi. Mycologist..

[17] Kotowski MA, Pietras M, Luczaj L. Extreme levels of mycophiliadocumented in Mazovia, a region of Poland. *J*. *Ethnobiol Etnomed*. 2019;15:12. Doi: https://doi.org/10.1186/s13002-019-0291-6. 10.1186/s13002-019-0291-6 PMC6371552 30755235

[18] INEGI (Instituto Nacional de Estadística Geografía e Informática). *Tlaxcala: XII Censo General de Población y Vivienda* 2000, Tomo 1. México City.

[19] INEGI (Instituto Nacional de Estadística Geografía e Informática). *Síntesis Geográfica de Tlaxcala* 1986. Mexico City.

[20] Romero CAT (1998). *Los temazcales de San Isidro Buensuceso*, *cultura*, *medicina y tradición de un pueblo tlaxcalteca*.

[21] Acosta PR, Delgado MJL, Cervantes SP. *La vegetación del estado de Tlaxcala*, *México*. *Jardín Botánico Tizatlán*. Tlaxcala: Gobierno del estado de Tlaxcala. Folleto de divulgación no. 6; 1991.

[22] INEGI (Instituto Nacional de Estadística Geografía e Informática). Censo de población y vivienda. 2010. Mexico City. http://www.inegi.org.mx/est/contenidos/proyectos/ccpv/cpv2010/default.aspx.

[23] Lara-Ponce EM, A Fernández, B Ramírez Valverde. 2002. Zentli. La agricultura del maíz en una comunidad nahua de la Malinche, Tlaxcala. 1 ed. CONACULTA. México. Pp 84.

[24] International Society of Ethnobiology (2006). International Society of Ethnobiology Code of Ethics (with 2008 additions). http://ethnobiology.net/code-of-ethics/. Acceced 05 Feb 2006.

[25] SOLAE 2015. http://asociacionetnobiologica.org.mx/aem/codigo-de-etica-de-solae. Accessed 28 Jan 2019.

[26] Weller SC. *Systematic Data Collection*. Sage Publications; 1988:96 pp.

[27] Estrada-Torres A, Aroche RM (1987). Acervo etnomicológico en tres localidades del municipio de Acambay, Estado de México. Rev Mex Mic.

[28] La E-TA (1989). *etnomicología: Avances*, *problemas y perspectivas*. Examen predoctoral.

[29] Reygadas-Prado F, Zamora-Martínez M, Cifuentes J (1995). Conocimiento sobre los hongos silvestres comestibles en las comunidades de Ajusco y Topilejo, D.F. Bol Soc Mex Mic..

[30] Montoya A, Estrada-Torres A, Caballero J (2002). Comparative ethnomycological survey of three localities from La Malinche volcano, México. J Ethnobiol.

[31] Lira-Franco N (2017). *Etnomicología de San Juan Tepulco*, *Municipio de Acajete*, *Puebla*.

[32] Martín del Campo R. Contribución al conocimiento de la nomenclatura náhuatl. *Bol Soc Mex Mic*. 1968;2:25-36.

[33] Kavalier-Smith T (2004). Only six kingdoms of life. Proc R Soc Lond B.

[34] Montoya A, Hernández-Totomoch O, Estrada-Torres A, Kong A (2003). Traditional knowledge about mushrooms in a Nahua community in the state of Tlaxcala, México. Mycologia.

[35] Graeme KA (2014). Mycetism: a review of the recent literature. J Med Toxicol.

[36] Montoya A, Méndez-Espinoza C, Flores-Rivera R, Kong A, Estrada-Torres A 2007. *Hongos tóxicos de Tlaxcala*. México City: Instituto Nacional de Investigaciones Forestales, Agrícolas y Pecuarias (INIFAP). Libro Técnico 2; 2007.

[37] Ramírez-Terrazo A (2017). *Importancia cultural de los hongos no comestibles en dos comunidades de las faldas del Volcán La Malintzi*, *Tlaxcala*.

[38] Bautista-González JA (2013). Conocimiento tradicional de hongos medicinales en seis localidades diferentes del país.

[39] Kong A, Montoya A, Estrada-Torres A, Fernández FJ, López DJ (2005). Hongos Macroscópicos. *Biodiversidad del Parque Nacional La Malinche (Tlaxcala*, *México)*.

[40] Torres-García EA. *Estudio ecológico y frecuencia de mención de los hongos silvestres en el Parque Nacional La Malinche*, *Tlaxcala*. Mexico City: Vachelor thesis. Universidad Nacional Autónoma de México; 2009.

[41] Jaime-Salinas M. *Etnomicología y taxonomía de hongos comestibles del género Lyophyllum (P*. *Karst) en Tlaxcala*.*México*: Graduate thesis. Universidad Autónoma de Tlaxcala; 2019.

[42] Martínez-Alfaro MA, Pérez-Silva E, Aguirre-Acosta CE (1983). Etnomicología y exploraciones micológicas en la Sierra Norte de Puebla. Bol Soc Mex Mic.

[43] Campos-Rivera M. *Adquisición del conocimiento etnobotánico en San Isidro Buensuceso*, *Tlaxcala*, *México*. Mexico City: Vachelor thesis. Universidad Nacional Autónoma de México; 2018.

[44] Alcorn JB (1981). Factors influencing botanical resource. Perception among the Huastec: suggestions for future ethnobotanical inquiry. J Ethnobiol.

[45] Turner NJ (1987). General plant categories in Thompson and Lillooet, two interior Salish languages of British Columbia. J Ethnobiol.

[46] Turner NJ (1989). “All berries have relations” mid-range folk plant groupings in Thompson and Lillooet interior Salish. J Ethnobiol.

[47] Johnson LM, Hargus S (1988). Classification and nomenclature in Witsuwit’en ethnobotany: a preliminary examination. J Ethnobiol.

[48] Johnson LM (1999). Gitksan plant classification and nomenclature. J Ethnobiol.

[49] Chacón S (1988). Conocimiento etnoecológico de los hongos en plan de palmar, Municipio de Papantla, Veracruz, México. Mic Neotrop Aplic.

[50] Ellen R (2008). Ethnomycology among the Nuaulu of the Moluccas: putting Berlin’s “general principles” of ethnobiological classification to the test. Econ Bot.

[51] Taylor PM (1990). The folk biology of the Tobelo people.

[52] Brown MF (1976). Is a rose a rose?. Cambridge Anthropologist.

[53] Laughlin RM (1975). The great Tzotzil dictionary of San Lorenzo Zinacantán.

[54] Escalante R (1982). Clasificación Matlalzinca de plantas y hongos.

[55] De Ávila A, Welden AL, Guzmán G (1980). Notes on the ethnomycology of Hueyapan, Morelos, México. J Ethnopharmacol.

[56] Rúan-Soto F (2017). 50 años de la etnomicología en México. Lacandonia.

[57] Becerril-Medina A. *Paralelismos y divergencias en la asignación de la nomenclatura tutunakú y científica de los hongos de Zongozotla*, *Puebla*, *México*. Mexico: Bachelor thesis. Universidad Nacional Autónoma de México; 2017.

[58] Montoya A, Briones-Dumas E, Núñez-López A, Kong A, Ortíz-Hernández AV, Moreno-Fuentes A (2019). Los hongos conocidos por la comunidad Yuhmu de Ixtenco, Tlaxcala. Scientia Fungorum.

[59] Alcántara-Salinas G, Hellen RF, Valiñas-Coalla L, Caballero-Nieto J, Argueta-Villamar A (2013). Alternative ways of representing Zapotec and Cuicatec folk classification of birds: a multidimensional model and its implications for culturally-informed conservation in Oaxaca, México. J Ethnobiol Etnomed.

